# Epidemiological characteristics of nasopharyngeal *Streptococcus pneumoniae* strains among children with pneumonia in Chongqing, China

**DOI:** 10.1038/s41598-019-40088-6

**Published:** 2019-03-01

**Authors:** Yi-Yi Yu, Xiao-Hong Xie, Luo Ren, Yu Deng, Yu Gao, Yao Zhang, Hui Li, Jian Luo, Zheng-Xiu Luo, En-Mei Liu

**Affiliations:** 1 Ministry of Education Key Laboratory of Child Development and Disorders; Key Laboratory of Pediatrics in Chongqing, CSTC2009CA5002; Chongqing International Science and Technology Cooperation Center for Child Development and Disorders, Children’s Hospital of Chongqing Medical University, Chongqing, 400014 China; 2 Department of Pediatrics, The Affiliated Hospital of Guizhou Medical University, Guiyang, 550004 China; 3 Department of Respiratory Medicine, Children´s Hospital of Chongqing Medical University, Chongqing, 400014 China

## Abstract

*Streptococcus pneumoniae* (pneumococcus) is the most common respiratory pathogen worldwide. Nasopharyngeal carriage with *S*. *pneumoniae* is the major source of lower respiratory tract infection and horizontal spread among children. Investigating nasopharyngeal *S*. *pneumoniae* is crucial for clinicians to control pneumococcus disease. Here, we retrospectively analyzed clinical information of 5,960 hospitalized children, focusing on pneumonia children less than five years with positive nasopharyngeal pneumococcal cultures. Nasopharyngeal aspirates (NPAs) were collected between June 2009 and December 2016, which were outside the pneumococcal conjugate vaccine(PCV) period. NPAs were subjected to common bacterial culture and antibiotic susceptibility tests, and serotypes were identified by both multiplex PCR and DNA sequencing. Results clearly revealed that clinical manifestations of the children whose NPAs were *S*. *pneumoniae* culture positive were serious, especially in those less than twelve months old. Fifteen different serotypes of nasopharyngeal *S*. *pneumoniae* were detected, the most common ones being 19F (35.2%), 6A/B (23.8%), 19A (11.4%), 15B/C (9.3%) and 23F (7.8%). Eight serotypes, accounting for 85.5% of the isolates, corresponded to the PCV13 serotypes. Approximately one-third of all *S*. *pneumoniae* strains were susceptible to penicillin. Overall, we consider nasopharyngeal *S*. *pneumoniae* culture is beneficial in assessing the situations of pneumonia children. Moreover, PCV13 could be useful in preventing pneumococcal disease in Chongqing, China.

## Introduction

*Streptococcus pneumonia* (pneumococcus) is a significant human pathogen that can cause pneumonia, otitis media, septicemia and meningitis, and constitutes an important cause of death among children under the age of five years^1^. Nasopharyngeal carriage with *S*. *pneumoniae* can be a reservoir of lower respiratory tract (LRT)infection, and is a major prerequisite towards the development of pneumococcal diseases^2–5^. The environment of the LRT is normally not sterile^6,7^; nasopharyngeal microbes can in fact be microaspirated in healthy individuals, and have a higher prevalence during seasons of respiratory diseases^8,9^. Ongoing surveillance of nasopharyngeal *S*. *pneumoniae* characteristics is, therefore, a significant source of epidemiological information. Nonetheless, data on nasopharyngeal *S*. *pneumoniae* strains in China have been limited, and here, we describe 7½ years (June 2009-December 2016) retrospective longitudinal study that characterized nasopharyngeal *S*. *pneumoniae* strains among a large cohort of pneumonia children. The study is based on *S*. *pneumoniae* bacterial culture, serotype distribution and antibiotic susceptibility. The characteristics of nasopharyngeal *S*. *pneumoniae* strains of pneumonia children are summarized here, which should be an important resource for clinicians as well as for local and national immunization programs.

## Results

### Prevalence and clinical correlates of nasopharyngeal *S*. *pneumoniae*

Series of clinical data were compared between nasopharyngeal *S*. *pneumoniae* culture-positive and -negative cohorts of a range of ages, grouped as less than 12 months (m), 13–36 m and 37–59 m (Table 1). Among these age groups, *S*. *pneumoniae* culture-positive rates were 13.9%, 20.4% and 18.9%, respectively. The following differences were observed, especially in children that were less than 12 m old. First, there were several factors associated with pneumococcal carriage and disease. They were more likely to have siblings (Chi-square test, *p* values were 0.019, 0.016 and 0.561 in the three age groups, respectively), histories of more than 5 days of prehospital antibiotic usage (Chi-square test, *p* = 0.002, 0.026, 0.222), repeated respiratory tract infection(RTI) (Chi-square test, *p* = 0.000, 0.37, 0.576), and the history of repeated wheezing (Chi-square test, *p* = 0.000, 0.042, 0.002). Second, the clinical manifestations were more serious in the positive groups. For example, the lengths of hospital stay were significantly longer in the positive groups (Mann-Whitney U test, *p* = 0.023, 0.013, 0.106). The morbidities of persistent or chronic pneumonia were more prevalent in the positive groups (Chi-square test, *p* = 0.000, 0.136, 0.277). Symptoms, such as fever (Chi-square test, *p* = 0.001, 0.992, 0.632) and wheeze (Chi-square test, *p* = 0.031, 0.004, 0.530), were also more severe in the positive groups. Third, inflammatory responses, both in the blood and the lungs, were more pronounced in the *S*. *pneumoniae*-positive groups, such as the counts of leukocyte (Mann-Whitney U test, *p* = 0.019, 0.005, 0.936), neutrophil (Mann-Whitney U test, *p* = 0.001, 0.756, 0.212)and thrombocyte(Mann-Whitney U test, *p* = 0.031, 0.254, 0.922). Another inflammatory marker, C-reactive protein (CRP), was also higher in positive groups (Chi-square test, *p* = 0.006, 0.148, 0.931).

**Table 1:** Comparison of clinical data between nasopharyngeal *S*. *pneumoniae* culture positive and negative groups among different ages.

Variables	*S*. *pneumoniae* (+)	*S*. *pneumoniae* (−)	P value
0–12 m (n = 1337)	n = 186 (13.9%)	n = 1151 (86.1%)	
**General Information**			
Male^a^	73.1 (66.1–79.3)	70.2 (67.5–72.8)	0.418
Premature History (≤36 week) ^a^	9.1 (5.4–14.2)	9.4 (7.8–11.2)	0.916
Siblings (n ≥ 1)^a^	42.5 (35.3–49.9)	33.6 (30.9–36.4)	***0***.***019***
Usage of Antibiotic (≥5 day)^*a^	39.8 (32.7–47.2)	28.4 (25.8–31.1)	***0***.***002***
History of Wheezing (≥3times)^a^	9.7 (5.8–14.9)	3.3 (2.4–4.5)	***0***.***000***
History of RTI (≥3 times)^a^	25.8 (19.7–32.7)	14.1 (12.1–16.2)	***0***.***000***
**Condition**			
Length of Stay (day)^b^	6 (6, 8)	6 (5, 8)	***0***.***023***
Persistent/Chronic^*a^	22 (16.3–28.7)	12.5 (10.7–14.6)	***0***.***000***
Severe^*a^	18.3 (13–24.6)	15.5 (13.4–17.7)	0.33
**Symptoms**			
Fever^a^	57.5 (50.1–64.7)	44.3 (41.4–47.2)	***0***.***001***
Wheeze^a^	60.2 (52.8–67.3)	51.7 (48.8–54.6)	***0***.***031***
Cough^a^	98.9 (96.2–99.9)	96.9 (95.7–97.8)	0.118
**Laboratory Parameters**			
Leukocyte (×10^9^/L)^b^	12 (9, 15.5)	11 (8.3, 14)	***0***.***019***
Neutrophil (%)^b^	38.5 (29, 52)	32 (24, 47)	***0***.***001***
Thrombocyte (×10^9^/L)^b^	450 (367, 558)	429 (331, 530)	***0***.***031***
CRP^*a^	14 (9.3–19.8)	7.8 (6.3–9.5)	***0***.***006***
**Imaging Features**			
Pleural Effusion^a^	2.2 (0.6–5.4)	0.7 (0.3–1.4)	0.073
Lobar Consolidation^a^	3.2 (1.2–6.9)	4.8 (3.6–6.2)	0.346
**13–36 m (n = 780)**	**n = 159 (20**.**4%)**	**n = 621 (79**.**6%)**	
**General Information**			
Male^a^	59.7 (51.7–67.4)	63.9 (60–67.7)	0.33
Premature History(≤36 week)^a^	10.1 (5.9–15.8)	8.5 (6.5–11)	0.545
Siblings(n ≥ 1)^a^	37.1 (29.6–45.1)	27.4 (23.9–31.1)	***0***.***016***
Usage of Antibiotic (≥5 day)^*a^	35.8 (28.4–43.8)	26.9 (23.4–30.6)	***0***.***026***
History of Wheezing (≥3times)^a^	14.5 (9.4–20.9)	9 (6.9–11.6)	***0***.***042***
History of RTI (≥3 times)^a^	37.1 (29.6–45.1)	33.3 (29.6–37.2)	0.37
**Condition**			
Length of Stay (day)^b^	6 (5, 8)	6 (5, 7)	***0***.***013***
Persistent/Chronic^*a^	12.6 (7.9–18.8)	8.7 (6.6–11.2)	0.136
Severe^*a^	9.4 (5.4–15.1)	12.4 (9.9–15.3)	0.301
**Symptoms**			
Fever^a^	74.8 (67.4–81.4)	74.9 (71.3–78.3)	0.992
Wheeze^a^	57.2 (49.2–65)	44.4 (40.5–48.5)	***0***.***004***
Cough^a^	97.5 (93.7–99.3)	96.9 (95.3–98.2)	1.000
**Laboratory Parameters**			
Leukocyte (×10^9^/L)^b^	11 (8.3, 14.5)	9.8 (7.3, 13.2)	***0***.***005***
Neutrophil (%)^b^	47 (34, 59.3)	45 (33, 59)	0.756
Thrombocyte (×10^9^/L)^b^	330 (249, 425.5)	317 (245, 407.8)	0.254
CRP^*a^	22 (15.8–29.3)	17.1 (14.2–20.3)	0.148
**Imaging Features**			
Pleural Effusion^a^	1.3 (0.2–4.5)	2.4 (1.4–4)	0.546
Lobar Consolidation^a^	8.2 (4.4–13.6)	8.5 (6.5–11)	0.885
**37–59 m (n = 238)**	**n = 45 (18**.**9%)**	**n = 193 (81**.**1%)**	
**General Information**			
Male^a^	62.2 (46.5–76.2)	49.2 (42–56.5)	0.116
Premature History (≤36 week)^a^	0	6.7 (3.6–11.2)	0.136
Siblings (n ≥ 1)^a^	22.2 (11.2–37.1)	26.4 (20.4–33.2)	0.561
Usage of Antibiotic (≥5 day)^*a^	37.8 (23.8–53.5)	28.5 (22.3–35.4)	0.222
History of Wheezing (≥3times)^a^	24.4 (12.9–39.5)	8.3 (4.8–13.1)	***0***.***002***
History of RTI (≥3 times)^a^	44.4 (29.6–60)	39.9 (32.9–47.2)	0.576
**Condition**			
Length of Stay (day)^b^	6.5 (5, 8)	6 (4, 7)	0.106
Persistent/Chronic^*a^	15.6 (6.5–29.5)	9.3 (5.6–14.3)	0.277
Severe^*a^	15.6 (6.5–29.5)	7.3 (4–11.9)	0.085
**Symptoms**			
Fever^a^	80 (65.4–90.4)	76.7 (70.1–82.5)	0.632
Wheeze^a^	26.7 (14.6–41.9)	22.3 (16.6–28.8)	0.530
Cough^a^	100 (92.1–100)	97.9 (94.8–99.4)	1.000
**Laboratory Parameters**			
Leukocyte (×10^9^/L)^b^	9.1 (7.1, 13)	9.1 (6.7, 13.1)	0.936
Neutrophil (%)^b^	59 (40.5, 67)	61 (44, 71)	0.212
Thrombocyte (×10^9^/L) ^b^	312.5 (213.8, 390.5)	299 (219.8, 390.8)	0.922
CRP^*a^	24.4 (12.9–39.5)	23.8 (18–30.5)	0.931
**Imaging Features**			
Pleural Effusion^a^	6.7 (1.4–18.3)	3.1 (1.2–6.6)	0.377
Lobar Consolidation^a^	26.7 (14.6–41.9)	9.3 (5.6–14.3)	***0***.***002***

Series of clinical data were compared between nasopharyngeal *S*. *pneumoniae* culture positive and negative cohorts of a range of ages. The conditions of children less than 5 years old were of utmost concern.

^a^The results were presented as percentages of the total (%) and 95% CI.

^b^The results were reported as median with IQR.

Usage of antibiotic^*^: days of antibiotic usage before NPAs collection.

Persistent/Chronic^*^: morbidities of persistent/chronic pneumonia.

Severe^*^: morbidities of severe pneumonia.

CRP^*^: the number of children whose CRP values were higher than normal range (8 mg/L).

P values < 0.05 were considered statistically significant in bold and italic.

Normal ranges of inflammation markers:

Leukocyte: 4–10 × 10^9^/L.

Neutrophil: 33–79%.

Thrombocyte: 100–300 × 10^9^/L.

### Distribution of nasopharyngeal *S*. *pneumoniae* serotypes

**Figure 1:**
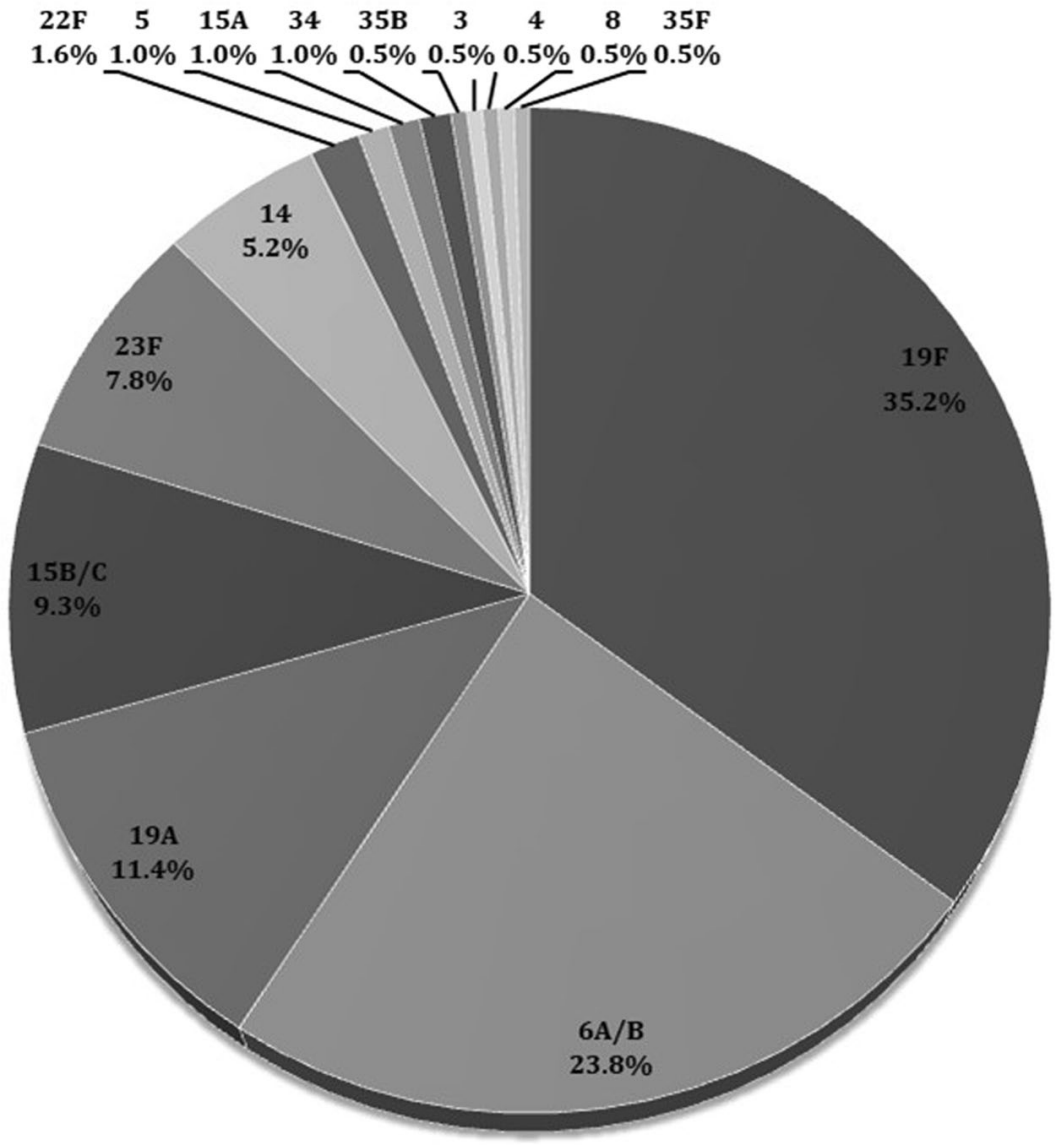
Distribution of nasopharyngeal *S*. *pneumoniae* serotypes among pneumonia children in Chongqing. Using multiplex PCR and DNA sequencing, fifteen different serotypes were identified among 193 nasopharyngeal *S*. *pneumoniae* culture positive pneumonia children. The most common serotypes were 19F(68/193, 35.2%), 6A/B(46/193, 23.8%), 19A(22/193, 11.4%), 15B/C(18/193, 9.3%), 23F(15/193, 7.8%) and 14(10/193, 5.2%), other serotypes were seldom detected. The table of serotypes distribution and the detected sequences have been provided in the online Supplementary Information file. Eight serotypes of PCV13 representing 85.5% were observed.

Using multiplex PCR and DNA sequencing, fifteen different serotypes were identified among 193 nasopharyngeal *S*. *pneumoniae* culture-positive pneumonia children whose demographic characteristics and clinical data were summarized in Table 2. The most common serotypes were 19 F(35.2%, 95% CI: 28.5–42.4), 6 A/B (23.8%, 95% CI: 18–30.5), 19 A(11.4%, 95% CI: 7.3–16.8), 15B/C(9.3%, 95% CI: 5.6–14.3), 23 F(7.8%, 95% CI: 4.4–12.5) and 14(5.2%, 95% CI: 2.5–9.3)(Fig. 1). The results clearly showed that 19 F and 6 A/B were the most common serotypes, accounting for more than half of all serotypes, whereas 19 A, 15B/C, 23 F and 14 were relatively less prevalent. The other serotypes were seldom detected in this study. All detected serotypes were validated by sequencing. The table of serotypes distribution (Supplementary Table 1) and the detected sequences are provided in the online Supplementary Information files. Currently, two PCVs are dominant: PCV10 and PCV13. In this study, six serotypes of PCV10 representing 73.6% (142/193, 95% CI: 67.4–79.8%) and eight serotypes of PCV13 representing 85.5% (165/193, 95% CI: 80.5–90.5%) were observed, respectively.

**Table 2:** The characteristics of 193 pneumonia children presenting with nasopharyngeal *S*. *pneumoniae* serotypes.

Variables	0–12 m (n = 103)	13–36 m (n = 72)	37–59 m (n = 18)
**General Information**			
Male^a^	74.8 (65.2–82.8)	62.5 (50.3–73.6)	55.6 (30.8–78.5)
Premature History (≤36 week)^a^	8.7 (4.1–15.9)	6.9 (2.3–15.5)	0
Siblings (n ≥ 1)^a^	39.8 (30.3–49.9)	41.7 (30.2–53.9)	27.8 (9.7–53.5)
Usage of Antibiotic (≥5 day)^*a^	38.8 (29.4–48.9)	47.2 (35.3–59.4)	38.9 (17.3–64.3)
History of Wheezing (≥3times)^a^	14.6 (8.4–22.9)	16.7 (8.9–27.3)	11.1 (1.4–34.7)
History of RTI (≥3 times)^a^	28.2 (19.7–37.9)	41.7 (30.2–53.9)	38.9 (17.3–64.3)
**Condition**			
Length of Stay (day)^b^	7 (6, 8)	6 (5, 8)	5.5 (5, 8)
Persistent/Chronic^*a^	28.2 (19.7–37.9)	13.9 (6.9–24.1)	11.1 (1.4–34.7)
Severe^*a^	15.5 (9.2–24)	8.3 (3.1–17.3)	11.1 (1.4–34.7)
**Symptoms**			
Fever^a^	67 (57–75.9)	70.8 (58.9–81)	61.1 (35.8–82.7)
Wheeze^a^	71.8 (62.1–80.3)	66.7 (54.6–77.3)	27.8 (9.7–53.5)
Cough^a^	100 (96.5–100)	100 (95–100)	100 (81.5–100)
**Laboratory Parameters**			
Leukocyte (×10^9^/L)^b^	12 (9.4, 16.7)	10.9 (8.8, 13.8)	7.5 (5.7, 11.5)
Neutrophil (%)^b^	37.5 (29.3, 52)	43 (34, 54)	49 (38.5, 64.8)
Thrombocyte (×10^9^/L)^b^	432 (359, 529)	361 (272, 458)	287.5 (214.3, 363.5)
CRP^*a^	14.6 (8.4–22.9)	15.3 (7.9–25.7)	22.2 (6.4–47.6)
**Imaging Features**			
Pleural Effusion^a^	1.9 (0.2–6.8)	2.8 (0.3–9.7)	5.6 (0.1–27.3)
Lobar Consolidation^a^	5.8 (2.2–12.3)	4.2 (0.9–11.7)	11.1 (1.4–34.7)

^a^The results were presented as percentages of the total (%) and 95% CI.

^b^The results were reported as median with IQR.

Usage of antibiotic^*^: days of antibiotic usage before NPAs collection.

Persistent/Chronic^*^: morbidities of persistent/chronic pneumonia.

Severe^*^: morbidities of severe pneumonia.

CRP^*^: the number of children whose CRP values were higher than normal range (8 mg/L).

Normal ranges of inflammation markers:

Leukocyte: 4–10 × 10^9^/L.

Neutrophil: 33–79%.

Thrombocyte: 100–300 × 10^9^/L.

### Antibiotic susceptibility of different *S*. *pneumoniae* serotypes

The antibiotic susceptibility tests were performed with nine classes of agents by the Kirby-Bauer disc diffusion method (Table 3). The outcomes were divided into susceptible, intermediate and resistant to specific antibiotics. The different antibiotics susceptibility of *S*. *pneumoniae* strains were summarized, which we generalize here. First, 63 (32.6%, 95% CI: 26.1–39.8) and 66 (34.2%, 95% CI: 27.5–41.4) of the 193 *S*. *pneumoniae* strains analyzed in the study were respectively susceptible and resistant to penicillin. Second, nearly all of the detected *S*. *pneumoniae* strains were susceptible to vancomycin(100%, 95% CI:98.1–100), linezolid(100%, 95% CI:98.1–100), levofloxacin(100%, 95% CI:98.1–100) and chloramphenicol(92.2%, 95% CI:87.5–95.6). Third, most of the *S*. *pneumoniae* strains were resistant to clindamycin (81.3%, 95% CI:75.1–86.6), tetracycline (88.6%, 95% CI:83.3–92.7), sulfamethoxazole(89.6%, 95% CI:84.5–93.6) and erythromycin(96.9%, 95% CI:93.4–98.8). 94.3% (182/193, 95% CI:90–97.1) of all strains in the NPAs were MDR strains in this study, and about 66.3% (128/193, 95% CI: 59.6–73%) of all strains were resistant to erythromycin, sulfamethoxazole, tetracycline and clindamycin simultaneously. No PDR *S*. *pneumoniae* strain has been detected so far. Lastly, antibiotic susceptibilities of different *S*. *pneumoniae* serotypes were summarized in Table 4. The penicillin resistance of 19F and 19A serotypes were similar, which might suggest that the 19 serogroup *S*. *pneumoniae* overall is more resistant to penicillin than other serogroups. Tables of antibiotic susceptibility presented as percentages of the total (%) and 95% CI were provided in the online Supplementary Information files (Supplementary Table 2 and Table 3)

**Table 3:** Antibiotic susceptibility of nasopharyngeal *S*. *pneumoniae* strains [n(%)].

	Total (n = 193)		
	Susceptible	Intermediate	Resistant
Vancomycin	193 (100)	0 (0)	0 (0)
Linezolid	193 (100)	0 (0)	0 (0)
Levofloxacin	193 (100)	0 (0)	0 (0)
Chloramphenicol	178 (92.2)	0 (0)	15 (7.8)
Penicillin	63 (32.6)	64 (33.2)	66 (34.2)
Clindamycin	16 (8.3)	20 (10.4)	157 (81.3)
Tetracycline	7 (3.6)	15 (7.8)	171 (88.6)
Sulfamethoxazole	12 (6.2)	8 (4.1)	173 (89.6)
Erythromycin	6 (3.1)	0 (0)	187 (96.9)

The antibiotic susceptibility tests were performed with nine classes of agents by the Kirby-Bauer disc diffusion method. The guidelines for classifying isolates as susceptible, intermediate or resistant were according to Clinical and Laboratory Standards Institute(CLSI).

**Table 4:** Antibiotic susceptibility of different *S*. *pneumoniae* serotypes [n(%)].

	19F (n = 68)			6A/B (n = 46)			19A (n = 22)			15B/C (n = 18)			23F (n = 15)		
	Sus-	Inter-	Res-	Sus-	Inter-	Res-	Sus-	Inter-	Res-	Sus-	Inter-	Res-	Sus-	Inter-	Res-
Vanco-	68 (100)	0 (0)	0 (0)	46 (100)	0 (0)	0 (0)	22 (100)	0 (0)	0 (0)	18 (100)	0 (0)	0 (0)	15 (100)	0 (0)	0 (0)
Linezo-	68 (100)	0 (0)	0 (0)	46 (100)	0 (0)	0 (0)	22 (100)	0 (0)	0 (0)	18 (100)	0 (0)	0 (0)	15 (100)	0 (0)	0 (0)
Levoflo-	68 (100)	0 (0)	0 (0)	46 (100)	0 (0)	0 (0)	22 (100)	0 (0)	0 (0)	18 (100)	0 (0)	0 (0)	15 (100)	0 (0)	0 (0)
Chlora-	64 (94.1)	0 (0)	4 (5.9)	38 (82.6)	0 (0)	8 (17.4)	21 (95.5)	0 (0)	1 (4.5)	18 (100)	0 (0)	0 (0)	15 (100)	0 (0)	0 (0)
Penici-	10 (14.7)	30 (44.1)	28 (41.2)	**20** (**43**.5)*	13 (28.3)	13 (28.3)	3 (13.6)	12 (54.5)	7 (31.8)	7 (38.9)^**a**^	3 (16.7)	8 (44.4)	6 (40)^**b**^	4 (26.7)	5 (33.3)
Clinda-	2 (2.9)	2 (2.9)	64 (94.1)	2 (4.3)	12 (26.1)	**32** (**69**.6)*	1 (4.5)	1 (4.5)	20 (90.9)	0 (0)	3 (16.7)	15 (83.3)	1 (6.7)	2 (13.3)	12 (80)
Sulfam-	2 (2.9)	1 (1.5)	65 (95.6)	2 (4.3)	1 (2.2)	43 (93.5)	2 (9.1)	0 (0)	20 (90.9)	0 (0)	1 (5.6)	17 (94.4)	0 (0)	0 (0)	15 (100)
Tetracy-	0 (0)	10 (14.7)	58 (85.3)	2 (4.3)	2 (4.3)	42 (91.3)	0 (0)	1 (4.5)	21 (95.5)	0 (0)	0 (0)	18 (100)	0 (0)	1 (6.7)	14 (93.3)
Erythr-	1 (1.5)	0 (0)	67 (98.5)	4 (8.7)	0 (0)	42 (91.3)	0 (0)	0 (0)	22 (100)	0 (0)	0 (0)	18 (100)	0 (0)	0 (0)	15 (100)

The differences of susceptible and resistant rates were compared among different serotypes.19 F serotype group was used as reference group.

Compared among multiply groups, p values were adjusted by Bonferroni method. So p values less than 0.0125 (0.05/4 = 0.0125) in bold and marked with * were statistically significant. ^a^p = 0.041; ^b^p = 0.035.

Abbreviations:

Sus-:susceptible, Inter-: intermediate, Res-:resistant.

Vanco-:vancomycin, Linezo-:linezolid, Levoflo-:levofloxacin, Chlora-:chloramphenicol, Penici-:penicillin, Clinda-:clindamycin, Sulfam-:sulfamethoxazole, Tetracy-: tetracycline, Erythr-: erythromycin.

## Discussion

About 800,000 children die each year due to pneumococcal disease^2^. As potential pathogen, *S*. *pneumoniae* can colonize the nasopharynx at low density without causing symptoms in healthy children, and are less likely to be detected by culture methods^10,11^. This study documented that positive *S*. *pneumoniae* culture of nasopharyngeal aspirates is an important reference for clinicians. *S*. *pneumoniae* culture-positive children had several specific characteristics, such as more than one siblings, history of repeated wheezing or respiratory tract infection (RTI) and more common antibiotic usage. Several previous studies had shown a clear association between siblings and the isolation of nasopharyngeal *S*. *pneumoniae*^12,13^. This is likely because close contact can transmit nasopharyngeal *S*. *pneumoniae* between siblings in the same family. Children with repeated wheezing were more likely to have positive nasopharyngeal *S*. *pneumoniae* detection, which is in agreement with other references^14,15^. Nasopharyngeal *S*. *pneumoniae* species appeared to contribute to respiratory symptoms^16^, and therefore, avoidance of exposure to *S*. *pneumoniae* pathogen or PCV inoculation should be beneficial for repeated RTI or wheezing in children^17^. As noted, the clinical manifestations of culture-positive children were obviously more serious than those of the negative ones, especially in younger children. The positive group not only had a longer recovery time, but also displayed higher levels of inflammatory markers than the negative group, which was consistent with previous reports^15,18^. These results supported *S*. *pneumoniae* carriage as a prerequisite for pneumococcal infection or diseases^5,19^. Nasopharyngeal colonization of *S*. *pneumoniae* resulted in increased numbers of mucosal and systemic inflammatory cells and higher concentrations of proinflammatory cytokines, which may impact on disease severity^20–22^. High bacterial load in the nasopharynx and local inflammatory reactions were indeed shown to be important in bacterial invasion of the LRT^23^. The above mentioned reasons may cause transmission of the nasopharyngeal *S*. *pneumoniae* into the LRT and aggravate the conditions of the children. Moreover, the younger children exhibited more serious clinical manifestations, probably due to their immature and weaker immunity. Taken together, these findings lead us to conclude that the children who tested positive for nasopharyngeal *S*. *pneumoniae* culture had certain risk factors and serious clinical manifestations, especially in children less than 12 m of age.

Various factors may promote or facilitate nasopharyngeal *S*. *pneumoniae* invasion of the LRT. Polysaccharide capsules, for example, may play a crucial role in the process^24,25^, which is not only significant for *S*. *pneumoniae* classification, but is also a cardinal determinant of vaccine target. Currently, according to the biochemical structures of the polysaccharide capsule and immunological distinction, *S*. *pneumoniae* can be divided into 48 serogroups and 97 serotypes^26^. The different serotypes have diverse characteristics, such as activation of complement, invasive ability and influence on biofilm formation^27,28^. It is believed that serotype epidemiology is quite variable both geographically and temporally. Prior to 2000, a large number of epidemiological studies reported that 19F, 6B, 23F and 14 serotypes accounted for the most common pneumococcal serotypes detected in the nasopharynx or in invasive diseases in the United States and several other countries^29^. Following the widespread use of PCVs, the incidence of pneumococcal diseases dramatically declined, bringing significant benefit to the developing countries^30–32^. PCVs not only protected the vaccinated individuals against disease but also reduced the carriage of vaccine serotypes that could induce herd effects across whole populations^33,34^. As shown in our study, 19F and 6A/B were the most common serotypes detected in nasopharynx in Chongqing while 19A, 15B/C, 23F, 14 and 22F were also detected, consistent with studies in other Chinese cities^35–38^. However, in these studies, *S*. *pneumoniae* strains were isolated from patients with invasive pneumococcal disease. These serotypes were mainly the PCV serotypes, likely because the PCVs had not yet been introduced in the national compulsory immunization program in China. Compared with the serotypes of nasopharyngeal *S*. *pneumoniae* strains detected in other studies^39^, there were some serotypes that were seldom detected or not detected in this study, such as serotypes 1, 3 and 5. Geographical division may well be a reason for this difference. Current United States guidelines on vaccine use recommend that children aged 2 to 59 m receive PCV13 as routine care^40^. Moreover, PCV13 covers serotypes of significantly higher invasive propensity, such as 1, 3, 5, 7F, and 19A^39,41^, of which 19A has exhibited high prevalence in China, as we have also shown. Furthermore, PCV13 covered the major proportion of serotypes in this study. We thus suggest that PCV13 could indeed be an effective strategy for prevention of invasive pneumococcal disease in Chongqing, and even nationally in China.

The rising occurrence of antibiotic resistance enables *S*. *pneumoniae* to be an alarming threat to children’s health. In fact, three major risk factors (antibiotic use, younger age and attending day-care facility) have been identified for nasopharyngeal-resistant *S*. *pneumoniae*^42^. Most importantly, association between carriage or infection with resistant *S*. *pneumoniae* and antibiotic use is now widely accepted. Many clinical studies have indeed linked the usage of antibiotics to community-wide antibiotic resistance^43,44^. It is now confirmed that antibiotic selection pressure enhances antibiotic resistance, and is linked to a reduction of susceptible bacterial strains, shift of the competitive balance, and dissemination of the existing resistant clone(s). The situation is particularly grave in China, where antibiotic usage is popular, as bacterial pathogens occur more frequently in developing countries. Reports have shown that a longer duration of carriage leads to higher incidence of resistance due to the greater risk of antibiotic exposure^45^. In the *S*. *pneumoniae* positive groups, over 30% of children received antibiotics for longer than 5 days before hospitalization, and thus, it is possible that the antibiotics contributed to the observed antibiotic resistance. As shown in our study, almost all *S*. *pneumoniae* strains were resistant to clindamycin, sulfamethoxazole, tetracycline and erythromycin, and the most common pattern was co-resistance to former four drugs. The results were consistent with previous reports^46,47^. Antibiotic-resistant *S*. *pneumoniae* could be detected if the antibiotic treatment is conducted within 4 weeks preceding the susceptibility test^12,48^. But these antibiotics have seldom been used for treatment in Chongqing for a long time. It was also reported that antibiotic resistance was due to the spread of strains belonging to a limited number of clones^49^. It was speculated that *S*. *pneumoniae* clones were stably resistant to former four antibiotics in Chongqing, which may be widely spread by over-use of antibiotics. And the detailed mechanisms of antibiotics resistance await further research. Conversely, *S*. *pneumoniae* strains have remained susceptible to other antibiotics, such as vancomycin, linezolid, levofloxacin and chloramphenicol, which lend hope to the treatment of resistant *S*. *pneumoniae*. Specifically, the former two antibiotics are better choices, while levofloxacin and chloramphenicol are cautiously used in children in the pediatric clinic. It was found that approximately one-third of all *S*. *pneumoniae* was susceptible to penicillin, which is also consistent with other studies^43^. Taken together, efforts to promote judicious antibiotic use in children appear to be the most appropriate measures to control the spread of antibiotic-resistant clones.

Lastly, we would like to point out potential limitations of our study. First, this study was conducted in a relatively isolated hospital population, and the mild pneumonia children that did not require hospitalization were, therefore, excluded. Second, this was a retrospective, single-center study, and thus, larger and continuous multicenter prospective studies are needed, which should provide crucial data to assess the national immunization program and the effects of vaccines and antibiotics on *S*. *pneumoniae* strains. Thirdly, serotyping NPAs were only possible for about 50% of all samples, as NPA samples with a DNA concentration lower than 20 ng/ul did not detected serotypes further. Finally, we have described the characteristics of the nasopharyngeal carriage *S*. *pneumoniae*, which should be valuable in monitoring *S*. *pneumoniae* epidemiology. Nonetheless, it may be more appropriate to collect *S*. *pneumoniae* from the lower respiratory tract to comprehensively monitor invasive *S*. *pneumoniae* characteristics in the future.

## Methods

### Ethics statement

The study was approved by the Ethics Committee of the Children’s Hospital of Chongqing Medical University (Permit number 2015–77). It was conducted in compliance with principles of the declaration of Helsinki. Informed consent was obtained from each parent or guardian on behalf of the children participants, prior to enrollment.

### Research subjects and sample collection

**Figure 2:**
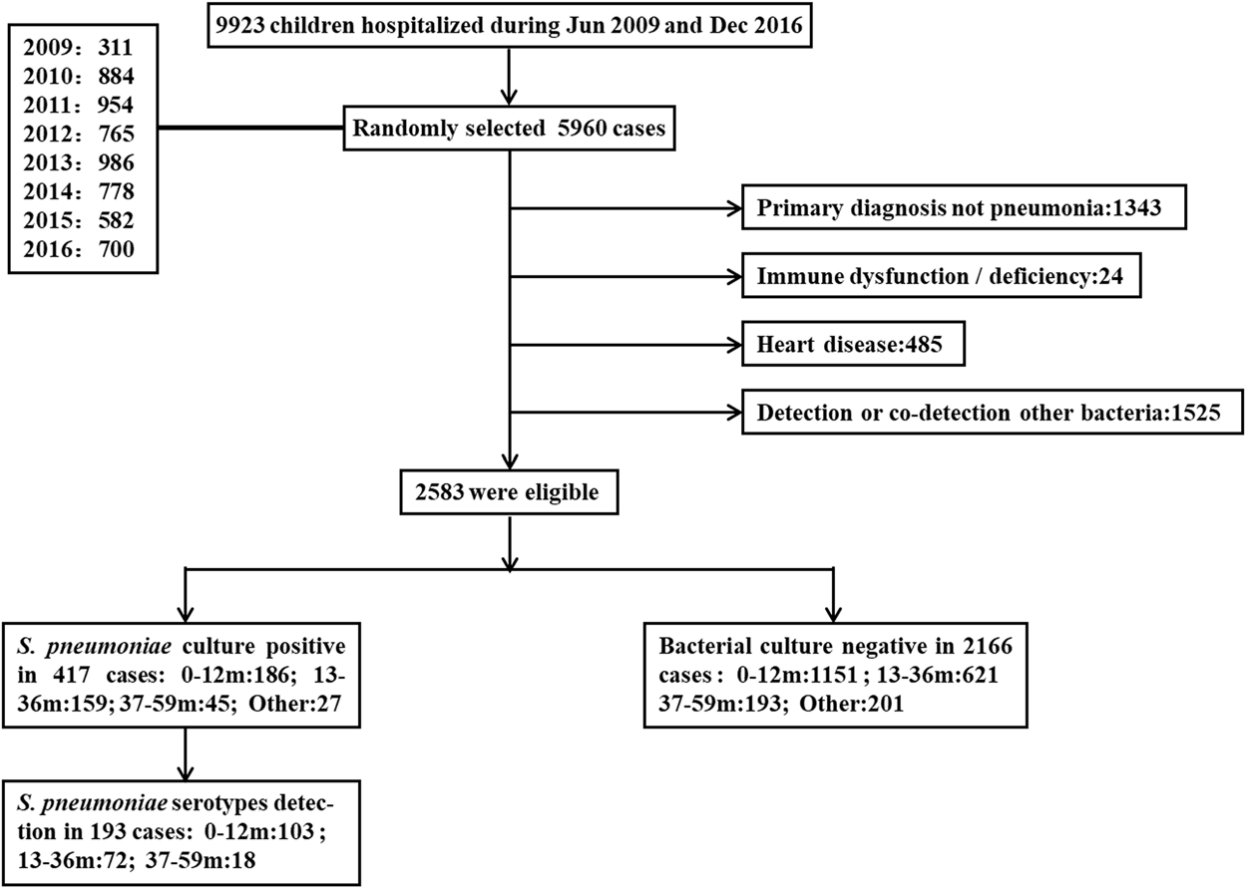
Flow chart of screening, eligibility and enrollment of children with pneumonia for comparison of nasopharyngeal *S*. *pneumoniae* culture. During the period from June 2009 and December 2016, a total of 9923 children with respiratory tract infection were hospitalized at the Department of Respiration in Children’s Hospital of Medical University of Chongqing, of which 5960 cases were randomly selected(minimum: 50 cases/month, 600 cases/ year). The numbers of NPA samples each year were also listed. 1343 cases with no pneumonia primary diagnosis, 24 cases with immune dysfunction/deficiency, 485 cases with heart disease and 1525 cases of bacterial common culture with detection or co-detection other bacteria were excluded sequentially. Lastly, 2583 cases were eligible, among which 417 cases were *S*. *pneumoniae* culture positive. The conditions of children less than 5 years old were of utmost concern, among which *S*. *pneumoniae* culture-positive rate was 16.6% (390/2355). They were further divided into three age groups: 0–12 m, 13–36 m and 37–59 m. NPAs with *S*. *pneumoniae* culture positive were not all detected serotypes. DNA concentrations of NPA samples more than 20 ng/μl were qualified. Finally, 193 NPAs were detected for *S*. *pneumoniae* serotypes further. The numbers of different age group children were shown. The primary diagnose not pneumonia contained: Upper respiratory airway infection: 124, Bronchitis:191, Bronchiolitis:514, Asthma:362, Other:152. Immune dysfunction/deficiency contained: primary immune deficiency or secondary immune deficiency/dysfunction:24. Heart disease contained: Atrial septal defect: 246, Interventricular septal defect:69, Patent ductus arteriosus:43, others:127. Detection or co-detection with other bacteria contained: Haemophilusparainfluenzae:359, Cardamorasia:174, Haemophilusinfluenzae:156, Escherichia coli:149, Staphylococcus aureus:129, Klebsiella pneumoniae:158, Others:191, co- detections:209.

During the period from June 2009 to December 2016, a total of 9923 children were hospitalized at the Department of Respiration in Children’s Hospital of Chongqing Medical University. A total of 5960 cases in this period were randomly selected and analyzed (minimum: 50 cases/month, 600 cases/year). In the primary diagnosis, children with no pneumonia were excluded. Pneumonia was diagnosed according to WHO clinical criteria^50^, lung auscultation with moist rales or evidence of patchy alveolar opacities on chest radiographs. Cases with immune dysfunction/immunodeficiency or heart disease were excluded sequentially. Cases that were positive for other nasopharyngeal bacteria by culture or which *S*. *pneumoniae* was co-detected with other bacteria were also excluded, so that the focus was mainly on nasopharyngeal *S*. *pneumoniae* strains. Overall, 2583 cases were eligible, consisting of positive nasopharyngeal *S*. *pneumoniae* culture in 417 cases and no bacteria in 2166 cases. The conditions of children less than 5 years old were of utmost concern. They were further divided into three age groups: 0–12 m, 13–36 m, and 37–59 m. The demographic and clinical information of the children were collected after admission. NPAs and venous blood were collected within 24 h by trained clinical personnel in accordance with standard protocols. Venous bloods were used for detection and quantification of inflammation markers, such as the leukocytes, neutrophil, thrombocyte and CRP. The normal ranges of these markers are listed in Table 1. Clinical criteria for diagnosis of severe pneumonia was defined by WHO on the basis of cough, tachypnea, difficult breathing, and general danger signs (central cyanosis, inability to breastfeed or drink, severe chest indrawing, head nodding, reduced level of consciousness and convulsions)^51^. Persistent or chronic pneumonia were defined as the course of pneumonia for 1–3 months or more than3 months, respectively. Chest radiographs were reviewed by specialists. NPA samples with a DNA concentration greater than 20 ng/ul were considered eligible, so only 193 NPA samples (193/390, 49.5%) from children under 5 years of age were further tested for S. pneumoniae serotypes. The screening, eligibility and enrollment of children with pneumonia are summarized in Fig. 2.

### NPA preparation

NPAs were collected into two tubes: one was immediately used for common bacteria culture and antibiotic susceptibility test by standard microbiological methods in the clinical bacteriology laboratory; the other one was sent to the respiratory laboratory for future analysis. The specimens were kept at 4 °C for a maximum of 4 h, and preserved at −80 °C until further use. DNA in the NPAs were extracted using a QIAamp DNA Mini Kit (Qiagen, Germany), following the manufacturer’s instructions. The concentrations of extracted DNA were then determined, and those exceeding 20 ng/ul were considered qualified. The DNA was preserved at −80 °C for subsequent tests.

### Bacterial culture and antibiotic susceptibility test

NPA specimens were inoculated on blood plates and chocolate plates within 2 hours of collection, and the plates were cultured at 35 °C for 24–48 hours in a 5–10% CO_2_ environment. *S*. *pneumoniae* was identified by colony morphology, gram staining, catalase test, optochin test, and biliary lysis test. Antibiotic sensitivity tests were performed using the Kirby-Bauer disc diffusion method to determine the sensitivity of all strains to vancomycin, linezolid, levofloxacin, chloramphenicol, penicillin, clindamycin, sulfamethoxazole, tetracycline, and erythromycin. Antibiotic susceptibility was determined according to the Clinical and Laboratory Standards Association (CLSI) guidelines of the year. *S*. *pneumoniae* ATCC49619 was included as the control strain. Multi-drug resistance (MDR) *S*. *pneumoniae* was defined as resistant to more than 3 classes of antibiotics, while pan-drug resistance (PDR) was defined as resistant to all antibiotics, including glycopeptides and linezolid.

### Multiplex PCR and sequencing

*S*. *pneumoniae* capsular serotypes were determined both by multiplex PCR and DNA sequencing. Twenty eight oligonucleotide primers, described previously^52^, were divided into 7 groups and used to detect *S*. *pneumoniae* serotypes (Supplementary Table 4), which included not only all common serotypes detected in China but also the PCV 13 serotypes. Multiplex PCR were performed in 25 μl volumes, each reaction mixture containing the following: 1 × PCR buffer (20 mM Tris-HCl, pH 8.0, 100mMKCl, 1 mM dithiothreitol, 0.1 mM EDTA, 0.5% Tween 20, 0.5% NonidetP-40), 6.25 μM of each deoxy nucleoside triphosphate, 62.5 μM of MgCl_2_, and 1.25 U of *Taq* DNA polymerase. All samples were analyzed using a commercial detection kit (TaKaRaEx Taq, RR01AM, Dalian, China and Applied Biosystems, Japan). The PCR parameters were: 95 °C for 5 min, followed by 35 amplification cycles of 95 °C for 45 s, 57 °C for 45 s, 72 °C for 1 min, and a final extension at 72 °C for 10 min. The PCR products were analyzed by electrophoresis in 2% NuSieve agarose gels. Specific target primers were then used for further amplification, and the products were sent for sequencing to the Beijing Genomics Institute (BGI).

### Statistical analysis

Continuous variables that do not satisfied the normal distribution were expressed as median with inter-quartile range(IQR);Categorical variables were reported as numbers (n), percentages of the total (%) and 95% confidence intervals (95% CI). Comparison between two groups, the Mann-Whitney U test and Chi-square test were used. Fisher’s exact tests were appropriately performed. Comparison among more than three groups, p values were adjusted by Bonferroni correction for multiple comparisons. All tests were two-sided considered statistically significant. SPSS (version 21.0) was used for all analyses.

## Supplementary information

supplementary file

## References

[1] O’Brien KL (2009). Burden of disease caused by Streptococcus pneumoniae in children younger than 5 years: global estimates. Lancet..

[2] Weiser JN (2010). The pneumococcus: why a commensal misbehaves. J Mol Med..

[3] Siegel SJ, Weiser JN (2015). Mechanisms of bacterial colonization of the respiratory tract. Annu Rev Microbiol..

[4] Kadioglu A, Weiser JN, Paton JC, Andrew PW (2008). The role of Streptococcus pneumoniae virulence factors in host respiratory colonization and disease. Nat Rev Microbiol..

[5] Bogaert D, De Groot R, Hermans PW (2004). Streptococcus pneumoniae colonisation: the key to pneumococcal disease. Lancet Infect Dis..

[6] Aho VTE (2015). The microbiome of the human lower airways: a next generation sequencing perspective. World Allergy Organ J..

[7] Segal LN, Rom WN, Weiden MD (2014). Lung microbiome for clinicians. New discoveries about bugs in healthy and diseased lungs. Ann Am Thorac Soc..

[8] Sullivan A, Hunt E, MacSharry J, Murphy DM (2016). The microbiome and the pathophysiology of asthma. Respir Res..

[9] Venkataraman A (2015). Application of a neutral community model to assess structuring of the human lung microbiome. MBio..

[10] Chien YW (2013). Density interactions among Streptococcus pneumoniae, Haemophilus influenzae and Staphylococcus aureus in the nasopharynx of young Peruvian children. Pediatr Infect Dis J..

[11] Da Gloria Carvalho M (2010). Revisiting pneumococcal carriage by use of broth enrichment and PCR techniques for enhanced detection of carriage and serotypes. J Clin Microbiol..

[12] Koliou MG (2018). Risk factors for carriage of Streptococcus pneumoniae in children. BMC Pediatrics..

[13] Toivonen L (2016). Burden of recurrent respiratory tract infections in children. J Pediatr Inf Dis..

[14] Esposito S (2016). Streptococcus pneumoniae colonisation in children and adolescents with asthma: impact of the heptavalent pneumococcal conjugate vaccine and evaluation of potential effect of thirteen-valent pneumococcal conjugate vaccine. BMC Infect Dis..

[15] Sohail I, Ghosh S, Mukundan S, Zelewski S, Khan MN (2018). Role of Inflammatory Risk Factors in the Pathogenesis of Streptococcus pneumoniae. Front immunol..

[16] Alsuwaidi AR (2018). Nasopharyngeal isolates and their clinical impact on young children with asthma: a pilot study. J Asthma Allergy..

[17] Cardozo DM (2008). Prevalence and risk factors for nasopharyngeal carriage of Streptococcus pneumoniae among adolescents. J Med Microbiol..

[18] Petraitiene S (2015). The influence of Streptococcus pneumoniae nasopharyngeal colonization on the clinical outcome of the respiratory tract infections in preschool children. BMC Infect Dis..

[19] Birgit S (2012). The fundamental link between pneumococcal carriage and disease. Expert Rev Vaccines..

[20] Wilson R (2015). Protection against Streptococcus pneumoniae lung infection after nasopharyngeal colonization requires both humoral and cellular immune responses. Mucosal Immunol..

[21] Yu D (2010). Impact of bacterial colonization on the severity, and accompanying airway inflammation, of virus-induced wheezing in children. Clin Microbiol Infect..

[22] Richards L, Ferreira DM, Miyaji EN, Andrew PW, Kadioglu A (2010). The immunising effect of pneumococcal nasopharyngeal colonisation; protection against future colonisation and fatal invasive disease. Immunobiology..

[23] Short KR, Reading PC, Wang N, Diavatopoulos DA, Wijburg OL (2012). Increased nasopharyngeal bacterial titers and local inflammation facilitate transmission of Streptococcus pneumoniae. MBio..

[24] Manso AS (2014). A random six-phase switch regulates pneumococcal virulence via global epigenetic changes. Nat Commun..

[25] Li J (2016). Epigenetic switch driven by DNA inversions dictates phase variation in Streptococcus pneumoniae. Plos Pathog..

[26] Geno KA (2015). Pneumococcal capsules and their types: Past, Present, and Future. Clin Microbiol Rev..

[27] Domenech M, Araujo-Bazan L, Garcia E, Moscoso M (2014). *In vitro* biofilm formation by Streptococcus pneumoniae as a predictor of post-vaccination emerging serotypes colonizing the human nasopharynx. Environ Microbiol..

[28] Kadioglu A (2002). Upper and lower respiratory tract infection by Streptococcus pneumoniae is affected by pneumolysin deficiency and differences in capsule type. Infect Immun.

[29] Johnson HL (2010). Systematic evaluation of serotypes causing invasive pneumococcal disease among children under five: the pneumococcal global serotype project. Plos Med..

[30] Kaplan SL (2004). Decrease of invasive pneumococcal infections in children among 8 children’s hospitals in the United States after the introduction of the 7-valent pneumococcal conjugate vaccine. Pediatrics..

[31] Harboe ZB (2010). Early effectiveness of heptavalent conjugate pneumococcal vaccination on invasive pneumococcal disease after the introduction in the Danish childhood immunization programme. Vaccine..

[32] Bettinger JA (2010). The effect of routine vaccination on invasive pneumococcal infections in Canadian children, Immunization Monitoring Program, Active 2000–2007. Vaccine..

[33] Collins AM (2015). First human challenge testing of a pneumococcal vaccine. Double-blind randomized controlled trial. Am J Respir Crit Care Med..

[34] Fitzwater SP, Chandran A, Santosham M, Johnson HL (2012). The worldwide impact of the seven-valent pneumococcal conjugate vaccine. Pediatr Infect Dis J..

[35] Pan F (2015). Serotype distribution, antimicrobial susceptibility, and molecular epidemiology of Streptococcus pneumoniae isolated from children in Shanghai, China. Plos One..

[36] Zhao C (2013). Phenotypic and genotypic characteristic of invasive pneumococcal isolates from both children and adult patients from a multicenter surveillance in China 2005–2011. PloS One..

[37] Li QH (2013). Spread of multidrug-resistant clonal complex 271 of serotype 19F Streptococcus pneumoniae in Beijing, China: characterization of serotype 19F. Epidemiol Infect..

[38] Kang LH (2016). Molecular epidemiology of pneumococcal isolates from children in China. Saudi Med J..

[39] Greenberg D (2017). Nasopharyngeal pneumococcal carriage during childhood community-acquired alveolar pneumonia:Relationship between specific serotypes and co-infecting viruses. J Infect Dis..

[40] Nuorti JP, Whitney CG (2010). Centers for Disease Conrol and Prevention(CDC). Prevention of pneumococcal disease among infantsand children — Use of 13-valent pneumococcal conjugate vaccine and 23-valent pneumococcal polysaccharide vaccine-Recommendations of the Advisory Committee on Immunization Practices (ACIP). MMWR Recomm Rep..

[41] Del Amo E (2014). High invasiveness of pneumococcal serotypes included in the new generation of conjugate vaccines. Clin Microbiol Infect..

[42] Belongia EA (2001). A community intervention trial to promote judicious antibiotic use and reduce penicillinresistant Streptococcus pneumoniae carriage in children. Pediatrics..

[43] Samore MH (2001). High rates of multiple antibiotic resistance in Streptococcus pneumoniae from healthy children living in isolated rural communities: Association with cephalosporin use and intrafamilial transmission. Pediatrics..

[44] Skalet AH (2010). Antibiotic selection pressure and macrolide resistance in nasopharyngeal Streptococcus pneumoniae: a cluster-randomized clinical trial. PLos Med..

[45] Lehtinen S (2017). Evolution of antibiotic resistance is linked to any genetic mechanism affecting bacterial duration of carriage. Proc Natl Acad Sci USA.

[46] Zhanel GG (2006). Molecular characterisation of Canadian paediatric multidrug-resistant Streptococcus pneumoniae from 1998–2004. Int J Antimicrob Agents..

[47] Nguyen QH (2010). Decreased Streptococcus pneumoniae susceptibility to oral antibiotics among children in rural Vietnam: a community study. BMC Infect Dis..

[48] Tyrstrup, M., Melander, E., Hedin, K., Beckman, A. & Molstad, S. Children with respiratory tract infections in Swedish primary care; prevalence of antibiotic resistance in common respiratory tract pathogens and relation to antibiotic consumption. *BMC Infect Dis*. **603** (2017). 10.1186/s12879-017-2703-3 PMC5583975 28870173

[49] Sjostrom K (2007). Clonal success of piliated penicillin nonsusceptible pneumococci. Proc Natl Acad Sci USA.

[50] Khan AJ, Khan JA, Akbar M, Addiss DG (1990). Acute respiratory infections in children: a case management intervention in Abbottabad District, Pakistan. Bulletin of the World Health Organization..

[51] WHO (2013). Hospital care for children: guidelines for the management of common illnesses with limited resources.

[52] Pai R, Gertz RE, Beall B (2006). Sequential multiplex PCR approach for determining capsular serotypes of Streptococcus pneumoniae isolates. J Clin Microbiol..

