# Supplementary material for: Epidemiological characteristics of nasopharyngeal Streptococcus pneumoniae strains among children with pneumonia in Chongqing, China
Source: Sci Rep. 2019 Mar 1;9:3324. doi: 10.1038/s41598-019-40088-6 (PMC6397308; doi:10.1038/s41598-019-40088-6)
Supplement: Supplementary file 1 — supplementary file [file 41598_2019_40088_MOESM1_ESM.docx]

**Epidemiological characteristics of** **nasopharyngeal *Streptococcus pneumoniae* strains among children with**

**pneumonia in Chongqing, China**

**Yi-Yi Yu ^1,2^, Xiao-Hong Xie ^1,3^, Luo Ren ^1^, Yu Deng^1,3^, Yu Gao ^1^, Yao Zhang ^1^, Hui Li^1^, Jian Luo^1,3^, Zheng-Xiu Luo^1,3^, En-Mei Liu^1,3,^***

^1^ Ministry of Education Key Laboratory of Child Development and Disorders; Key Laboratory of Pediatrics in Chongqing, CSTC2009CA5002; Chongqing International Science and Technology Cooperation Center for Child Development and Disorders, Children's Hospital of Chongqing Medical University, Chongqing 400014, China

^2^ Department of Pediatrics, The Affiliated Hospital of Guizhou Medical University, Guiyang, 550004, China

^3^ Department of Respiratory Medicine, Children´s Hospital of Chongqing Medical University, Chongqing, 400014, China

Supplementary table 1: Distribution of nasopharyngeal *S. pneumoniae* serotypes among pneumonia children in Chongqing

| Serotypes | n | % (95%CI) |
| --- | --- | --- |
| 19F | 68 | 35.2 (28.5-42.4) |
| 6A/B | 46 | 23.8 (18-30.5) |
| 19A | 22 | 11.4 (7.3-16.8) |
| 15B/C | 18 | 9.3 (5.6-14.3) |
| 23F | 15 | 7.8 (4.4-12.5) |
| 14 | 10 | 5.2 (2.5-9.3) |
| 22F | 3 | 1.6 (0.3-4.5) |
| 5 | 2 | 1 (0.1-3.7) |
| 15A | 2 | 1(0.1-3.7) |
| 34 | 2 | 1(0.1-3.7) |
| 35B | 1 | 0.5(0-2.9) |
| 3 | 1 | 0.5(0-2.9) |
| 4 | 1 | 0.5(0-2.9) |
| 8 | 1 | 0.5(0-2.9) |
| 35F | 1 | 0.5(0-2.9) |
| Total | 193 | 100 |

Supplementary table 2: Antibiotic susceptibility of nasopharyngeal *S. pneumoniae* strains [%(95%CI)]

|  | **Susceptible** | | **Intermediate** | **Resistant.** |
| --- | --- | --- | --- | --- |
| **Vancomycin** | 100(98.1-100) | | 0 | 0 |
| **Linezolid** | 100(98.1-100) | | 0 | 0 |
| **Levofloxacin** | 100(98.1-100) | | 0 | 0 |
| **Chloramphenicol** | 92.2(87.5-95.6) | | 0 | 7.8(4.4-12.5) |
| **Penicillin** | 32.6(26.1-39.8) | | 33.2(26.6-40.3) | 34.2(27.5-41.4) |
| **Clindamycin** | 8.3(4.8-13.1) | | 10.4(6.5-15.6) | 81.3(75.1-86.6) |
| **Tetracycline** | 3.6(1.5-7.3) | | 7.8(4.4-12.5) | 88.6(83.3-92.7) |
| **Sulfamethoxazole** | 6.2(3.3-10.6) | | 4.1(1.8-8) | 89.6(84.5-93.6) |
| **Erythromycin** | 3.1(1.2-6.6) | | 0 | 96.9(93.4-98.9) |
|  |  |  |  |  |

The antibiotic susceptibility tests were performed with nine classes of agents by the Kirby-Bauer disc diffusion method. The guidelines for classifying isolates as susceptible, intermediate or resistant were according to Clinical and Laboratory Standards Institute(CLSI).

The results were presented as percentages of the total (%) and 95%CI.

Supplementary table 3: Antibiotic susceptibility of different

*S. pneumoniae* serotypes [%(95%CI)]

|  | **Susceptible** | **Intermediate** | **Resistant.** |
| --- | --- | --- | --- |
| **19F** |  | | |
| **Vancomycin** | 100(94.7-100) | 0 | 0 |
| **Linezolid** | 100(94.7-100) | 0 | 0 |
| **Levofloxacin** | 100(94.7-100) | 0 | 0 |
| **Chloramphenicol** | 94.1(85.6-98.4) | 0 | 5.9(1.6-14.4) |
| **Penicillin** | 14.7(7.3-25.4) | 44.1(32.1-56.7) | 41.2(29.4-53.8) |
| **Clindamycin** | 2.9(0.4-10.2) | 2.9(0.4-10.2) | 94.1(85.6-98.4) |
| **Tetracycline** | 2.9(0.4-10.2) | 1.5(0.04-7.9) | 95.6(87.6-99.1) |
| **Sulfamethoxazole** | 0 | 14.7(7.3-25.4) | 85.3(74.6-92.7) |
| **Erythromycin** | 1.5(0.04-7.9) | 0 | 98.5(92.1-100) |
| **6A/B** |  | | |
| **Vancomycin** | 100(92.3-100) | 0 | 0 |
| **Linezolid** | 100(92.3-100) | 0 | 0 |
| **Levofloxacin** | 100(92.3-100) | 0 | 0 |
| **Chloramphenicol** | 82.6(68.6-92.2) | 0 | 17.4(7.8-31.4) |
| **Penicillin** | 43.5(28.9-58.9) | 28.3(16-43.5) | 28.3(16-43.5) |
| **Clindamycin** | 4.3(0.5-14.8) | 26.1(14.3-41.1) | 69.6(54.3-82.3) |
| **Tetracycline** | 4.3(0.5-14.8) | 2.2(0.1-11.5) | 93.5(82.1-98.6) |
| **Sulfamethoxazole** | 4.3(0.5-14.8) | 4.3(0.5-14.8) | 91.3(79.2-97.6) |
| **Erythromycin** | 8.7(2.4-20.8) | 0 | 91.3(79.2-97.6) |
| **19A** |  | | |
| **Vancomycin** | 100(84.6-100) | 0 | 0 |
| **Linezolid** | 100(84.6-100) | 0 | 0 |
| **Levofloxacin** | 100(84.6-100) | 0 | 0 |
| **Chloramphenicol** | 95.5(77.2-99.9) | 0 | 4.5(0.1-22.8) |
| **Penicillin** | 13.6(2.9-34.9) | 54.5(32.2-75.6) | 31.8(13.9-54.9) |
| **Clindamycin** | 4.5(0.1-22.8) | 4.5(0.1-22.8) | 90.9(70.8-98.9) |
| **Tetracycline** | 9.1(1.1-29.2) | 0 | 90.9(70.8-98.9) |
| **Sulfamethoxazole** | 0 | 4.5(0.1-22.8) | 95.5(77.2-99.9) |
| **Erythromycin** | 0 | 0 | 100(84.6-100) |
| **15B/C** |  | | |
| **Vancomycin** | 100(81.5-100) | 0 | 0 |
| **Linezolid** | 100(81.5-100) | 0 | 0 |
| **Levofloxacin** | 100(81.5-100) | 0 | 0 |
| **Chloramphenicol** | 100(81.5-100) | 0 | 0 |
| **Penicillin** | 38.9(17.3-64.3) | 16.7(3.6-41.4) | 44.4(21.5-69.2) |
| **Clindamycin** | 0 | 16.7(3.6-41.4) | 83.3(58.6-96.4) |
| **Tetracycline** | 0 | 5.6(0.1-27.3) | 94.4(72.7-99.9) |
| **Sulfamethoxazole** | 0 | 0 | 100(81.5-100) |
| **Erythromycin** | 0 | 0 | 100(81.5-100) |
| **23F** |  | | |
| **Vancomycin** | 100(78.2-100) | 0 | 0 |
| **Linezolid** | 100(78.2-100) | 0 | 0 |
| **Levofloxacin** | 100(78.2-100) | 0 | 0 |
| **Chloramphenicol** | 100(78.2-100) | 0 | 0 |
| **Penicillin** | 40(16.3-67.7) | 26.7(7.8-55.1) | 33.3(11.9-61.6) |
| **Clindamycin** | 6.7(0.2-32) | 13.3(1.7-40.5) | 80(51.9-95.7) |
| **Tetracycline** | 0 | 0 | 100(78.2-100) |
| **Sulfamethoxazole** | 0 | 6.7(0.2-32) | 93.3(68.1-99.8) |
| **Erythromycin** | 0 | 0 | 100(78.2-100) |

For each serotype, the outcomes were divided into susceptible, intermediate or resistant.

The results were presented as percentages of the total (%) and 95%CI.

Supplementary table 4: Serotype detection process by multiplex PCR

| **Reaction** | **Serotypes** |
| --- | --- |
| mPCR 1 | 19F,6A/B,15B/C,22F |
| mPCR 2 | 1,15A,19A,23F |
| mPCR 3 | 4,5,9V,14 |
| mPCR 4 | 3,10A,34,35F |
| mPCR 5 | 12F,16F,18,35B |
| mPCR 6 | 7C,8,17F,20 |
| mPCR 7 | 7F,11A,31,33F |

Supplementary serotypes sequences:

**6A/B**

>CQ81-Jul2009

GCGATTGTTATTATGTCCGTGTCTTCGATACAAGACCAGTTGCTCAGGGCAGAACAACACCTTCCCATACTCTAGTGCAAACTTTGCAAAATAATTATCATGAGACAGTTTTTCTATATATAAATAATCTTTATCAATATCAACTGAAGGAACCATCTCTAACAATGCATGATTAAAGCCTGCCGTACACCCCCAGATATAGGCATGAATAAAATACAAAT

>CQ200-Sep2009

CGGGGGGTCGGCAGGCTTTATCATGCATTGTTAGAGATGGTTCCTTCAGTTGATATTGATAAAGATTATTTATATATAGAAAAACTGTCTCATGATAATTATTTTGCAAAGTTTGCACTAGAGTATGGGAAGGTGTTGTTCTGCCCTGAGCAACTGGTCTTGTATCGAAGACACGGACATAATGTAACAACTAGTCATCATTTTAAATTATCTCCGCTAAA

>CQ288-Dec2009

TTAATCATGCATTGTTAGAGATGGTTCCTTCAGTTGATATTGATAAAGATTATTTATATATAGAAAAACTGTCTCATGATAATTATTTTGCAAAGTTTGCACTAGAGTATGGGAAGGTGTTGTTCTGCCCTGAGCAACTGGTCTTGTATCGAAGACACGGACATAATGTAACAACTAGTCATCATTTTAAATTATCTCCGCTAAA

>CQ649-Jul2010

AGGGGGGGGTCTGCAGGTTTTATCATGCATTGCTAGAGATGGTTCCTTCAGTTGATATTGATAAAGATTATTTATATATAGAAAAACTGGCTCATGATAGTTATTTTGCAAAGTTTGCACTAGAGTATGGGAAGGTGTTGTTCTGCCCTGAGCAACTGGTCTTGTATCGAAGACATGGACATAATGTAACAACTAGTCATCATTTTAAATTATCTCCGCTAAA

>CQ678-Jul2010

AGGGAGTCTGCAGGTTTTATCATGCATTGCTAGAGATGGTTCCTTCAGTTGATATTGATAAAGATTATTTATATATAGAAAAACTGTCTCATGATAATTATTTTGCAAAGTTTGCACTAGAGTATGGGAAGGTGTTGTTCTGCCCTGAGCAACTGGTCTTGTATCGAAGACATGGACATAATGTAACAACTAGTCATCATTTTAAATTATCTCCGCTAAA

>CQ3244-Apr2012

ATGGGGGGGGTCGGCAGGCTTTATCATGCATTGTTAGAGATGGTTCCTTCAGTTGATATTGATAAAGATTATTTATATATAGAAAAACTGTCTCATGATAATTATTTTGCAAAGTTTGCACTAGAGTATGGGAAGGTGTTGTTCTGCCCTGAGCAACTGGTCTTGTATCGAAGACACGGACATAATGTAACAACTAGTCATCATTTTAAATTATCTCCGCTAAA

>CQ3263-Apr2012

GTGGGGCACTGACTGCTGTACTGCACATTGCTAGAGATGGTTCCTTCAGTTGATATTGATAAAGATTATTTATATATAGAAAAACTGGCTCATGATAGTTATTTTGCAAAGTTTGCACTAGAGTATGGGAAGGTGTTGTTCTGCCCTGAGCAACTGGTCTTGTATCGAAGACATGGACATAATGTAACAACTAGTCATCATTTTAAATTATCTCCGCTAAA

>CQ3298-Apr2012

CGGCTGTTATTATGTCATGTCTTCGATACAAGACCAGTTGCTCAGGGCAGAACAACACCTTCCCATACTCTAGTGCAAACTTTGCAAAATAACTATCATGAGCCAGTTTTTCTATATATAAATAATCTTTATCAATATCAACTGAAGGAACCATCTCTAGCAATGCATGATTAAAACCTGCAGTACACCCCCAGATATAGGCATGAATAAAATACAAATTAATG

>CQ3615-Jul2012

GTGACTGTTCTTATGTACATGTCTTCGATACAAGACCAGTTGCTCAGGGCAGAACAACACCTTCCCATACTCTAGTGCAAACTTTGCAAAATAACTATCATGAGCCAGTTTTTCTATATATAAATAATCTTTATCAATATCAACTGAAGGAACCATCTCTAGCAATGCATGATTAAAACCTGCAGTACACCCCCAGATATAGGCATGAATAAAATACAAATTA

>CQ4007-Nov2012

GGCCCCCTTCCTTCTCATTTTTAATTATTTGCGCTAGATGGTTCCTTCAGTTGATATTGATAAAGATTATTTATATATAGAAAAACTGGCTCATGATAGTTATTTTGCAAAGTTTGCACTAGAGTATGGGAAGGTGTTGTTCTGCCCTGAGCAACTGGTCTTGTATCGAAGACATGGACATAATGTAACAACTAGTCATCATTTTAAATTATCTCCGCTAAA

>CQ4508-Mar2013

GGGGGGGGGTCGGGAGGCTTTATCATGCATTGTTAGAGATGGTTCCTTCAGTTGATATTGATAAAGATTATTTATATATAGAAAAACTGTCTCATGATAATTATTTTGCAAAGTTTGCACTAGAGTATGGGAAGGTGTTGTTCTGCCCTGAGCAACTGGTCTTGTATCGAAGACACGGACATAATGTAACAACTAGTCATCATTTTAAATTATCTCCGCTAAA

>CQ5606-Oct2013

GGGGGGGTCTGCAGGTTTTATCATGCATTGCTAGAGATGGTTCCTTCAGTTGATATTGATAAAGATTATTTATATATAGAAAAACTGGCTCATGATAGTTATTTTGCAAAGTTTGCACTAGAGTATGGGAAGGTGTTGTTCTGCCCTGAGCAACTGGTCTTGTATCGAAGACATGGACATAATGTAACAACTAGTCATCATTTTAAATTATCTCCGCTAAA

>CQ6662-May2014

ACGGGGTCGGCAGGCTTTATCATGCATTGTTAGAGATGGTTCCTTCAGTTGATATTGATAAAGATTATTTATATATAGAAAAACTATCTCATGATAATTATTTTGCAAAGTTTGCACTAGAGTATGGGAAGGTGTTGTTCTGCCCTGAGCAACTGGTCTTGTATCGAAGACACGGACATAATGTAACAACTAGTCATCATTTTAAATATTCTCCGCTAA

>CQ6669-May2014

TAGGGGTCTGCACGTTTTATCATGCATTGCTAGAGATGGTTCCTTCAGTTGATATTGATAAAGATTATTTATATATAGAAAAACTGTCTCATGATAATTATTTTGCAAAGTTTGCACTAGAGTATGGGAAGGTGTTGTTCTGCCCTGAGCAACTGGTCTTGTATCGAAGACATGGACATAATGTAACAACTAGTCATCATTTTAAATTATCTCCGCTAAA

>CQ6712-Jun2014

GGGGGGGTGTCTGCAGGGTTTTATCATGCATTGCTAGAGATGGTTCCTTCAGTTGATATTGATAAAGATTATTTATATATAGAAAAACTGGCTCATGATAGTTATTTTGCAAAGTTTGCACTAGAGTATGGGAAGGTGTTGTTCTGCCCTGAGCAACTGGTCTTGTATCGAAGACATGGACATAATGTAACAACTAGTCATCATTTTAAATTATCTCCGCTAAA

>CQ6911-Jul2014

GGGGGGTTTGGCCGGGCCTCGTTTTAATTATCTGCGTTTCTGGTTCCTTCAGTTGATATTGATAAAGATTATTTATATATAGAAAAACTGGCTCATGATAGTTATTTTGCAAAGTTTGCACTAGAGTATGGGAAGGTGTTGTTCTGCCCTGAGCAACTGGTCTTGTATCGAAGACATGGACATAATGTAACAACTAGTCATCATTTTAAATTATCTCCGCTAAA

>CQ6984-Jul2014

TTGGGGGTCCTGCAGGTTTTATCATGCATTGCTAGAGATGGTTCCTTCAGTTGATATTGATAAAGATTATTTATATATAGAAAAACTGGCTCATGATAGTTATTTTGCAAAGTTTGCACTAGAGTATGGGAAGGTGTTGTTCTGCCCTGAGCAACTGGTCTTGTATCGAAGACATGGACATAATGTAACAACTAGTCATCATTTTAAATTATCTCCGCTAAA

>CQ7023-Aug2014

AGGGGCGGTCGGGCAGGCTTTATCATGCATTGTTAGAGATGGTTCCTTCAGTTGATATTGATAAAGATTATTTATATATAGAAAAACTGTCTCATGATAATTATTTTGCAAAGTTTGCACTAGAGTATGGGAAGGTGTTGTTCTGCCCTGAGCAACTGGTCTTGTATCGAAGACACGGACATAATGTAACAACTAGTCATCATTTTAAATTATCTCCGCTAAA

>CQ9409-May2016

GGGGAGTCGGCAGGCTTTATCATGCATTGTTAGAGATGGTTCCTTCAGTTGATATTGATAAAGATTATTTATATATAGAAAAACTGTCTCATGATAATTATTTTGCAAAGTTTGCACTAGAGTATGGGAAGGTGTTGTTCTGCCCTGAGCAACTGGTCTTGTATCGAAGACACGGACATAATGTAACAACTAGTCATCATTTTAAATTATCTCCGCTAAA

>CQ8192-Apr2015

TGGGGGGGCCTGGCAGGTTTTAATCATGCATTGCTAGAGATGGTTCCTTCAGTTGATATTGATAAAGATTATTTATATATAGAAAAACTGGCTCATGATAGTTATTTTGCAAAGTTTGCACTAGAGTATGGGAAGGTGTTGTTCTGCCCTGAGCAACTGGTCTTGTATCGAAGACATGGACATAATGTAACAACTAGTCATCATTTTAAATTATCTCCGCTAAA

>CQ8230-Apr2015

GGGGCGGGTCTGCAGGTTTTATCATGCATTGCTAGAGATGGTTCCTTCAGTTGATATTGATAAAGATTATTTATATATAGAAAAACTGGCTCATGATAGTTATTTTGCAAAGTTTGCACTAGAGTATGGGAAGGTGTTGTTCTGCCCTGAGCAACTGGTCTTGTATCGAAGACATGGACATAATGTAACAACTAGTCATCATTTTAAATTATCTCCGCTAAA

>CQ8398-Jun2015

TTAATCATGCATTGTTAGAGATGGTTCCTTCAGTTGATATTGATAAAGATTATTTATATATAGAAAAACTGTCTCATGATAATTATTTTGCAAAGTTTGCACTAGAGTATGGGAAGGTGTTGTTCTGCCCTGAGCAACTGGTCTTGTATCGAAGACACGGACATAATGTAACAACTAGTCATCATTTTAAATTATCTCCGCTAAA

>CQ8990-Jan2016

GGGGGGGGTCTGCAGGGTTTTATCATGCATTGCTAGAGATGGTTCCTTCAGTTGATATTGATAAAGATTATTTATATATAGAAAAACTGGCTCATGATAGTTATTTTGCAAAGTTTGCGCTAGAGTATGGGAAGGTGTTGTTCTGCCCTGAACAACTGGTCTTGTATCGAAGACATGGACATAATGTAACAACTAGTCATCATTTTAAATTATCTCCGCTAAA

>CQ9149-Feb2016

GGGGGTCGGCAGGCTTTATCATGCATTGTTAGAGATGGTTCCTTCAGTTGATATTGATAAAGATTATTTATATATAGAAAAACTGTCTCATGATAATTATTTTGCAAAGTTTGCACTAGAGTATGGGAAGGTGTTGTTCTGCCCTGAGCAACTGGTCTTGTATCGAAGACACGGACATAATGTAACAACTAGTCATCATTTTAAATTATCTCCGCTAAA

>CQ9134-Feb2016

GGCCGTCTGCAGGTTTTATCATGCATTGCTAGAGATGGTTCCTTCAGTTGATATTGATAAAGATTATTTATATATAGAAAAACTGGCTCATGATAGTTATTTTGCAAAGTTTGCACTAGAGTATGGGAAGGTGTTGTTCTGCCCTGAGCAACTGGTCTTGTATCGAAGACATGGACATAATGTAACAACTAGTCATCATTTTAAATTATCTCCGCTAAA

>CQ6898-Jul2014

CTGGCACGCTTTTATCATGCTTTGCGCTAGATGGTTCCTTCAGTTGATATTGATAAAGATTATTTATATATAGAAAAACTGGCTCATGATAGTTATTTTGCAAAGTTTGCACTAGAGTATGGGAAGGTGTTGTTCTGCCCTGAGCAACTGGTCTTGTATCGAAGACATGGACATAATGTAACAACTAGTCATCATTTTAAATTATCTCCGCTAAAAA

>CQ7799-Jan2015

CTGAGTCCTGCATGTTTTATCATGCATTGCTAGAGATGGTTCCTTCAGTTGATATTGATAAAGATTATTTATATATAGAAAAACTGGCTCATGATAGTTATTTTGCAAAGTTTGCACTAGAGTATGGGAAGGTGTTGTTCTGCCCTGAGCAACTGGTCTTGTATCGAAGACATGGACATAATGTAACAACTAGTCATCATTTTAAATTATCTCCGCTAAA

>CQ1400-Feb2011

CATTGGTCTTGTTAGTCTCAGTCATGCTTTGCTCCACAAAGTTCCTTCAGTTGATATTGATAAAGATTATTTATATATAGAAAAACTGGCTCATGATAGTTATTTTGCAAAGTTTGCACTAGAGTATGGGAAGGTGTTGTTCTGCCCTGAGCAACTGGTCTTGTATCGAAGACATGGACATAATGTAACAACTAGTCATCATTTTAAATTATCTCCGCTAAA

>CQ4697-Apr2013

CGGTCGTCTTGCACGTTTTTATCATGCATTGCTAGAGATGGTTCCTTCAGTTGATATTGATAAAGATTATTTATATATAGAAAAACTGGCTCATGATAGTTATTTTGCAAAGTTTGCACTAGAGTATGGGAAGGTGTTGTTCTGCCCTGAGCAACTGGTCTTGTATCGAAGACATGGACATAATGTAACAACTAGTCATCATTTTAAATTATCTCCGCTAAA

>CQ9385-May2016

GGGAGGTCTGCAGGTTTTATCATGCATTGCTAGAGATGGTTCCTTCAGTTGATATTGATAAAGATTATTTATATATAGAAAAACTGGCTCATGATAGTTATTTTGCAAAGTTTGCACTAGAGTATGGGAAGGTGTTGTTCTGCCCTGAGCAACTGGTCTTGTATCGAAGACATGGACATAATGTAACAACTAGTCATCATTTTAAATTATCTCCGCTAAA

>CQ220-Oct2009

TTAATCATGCATTGTTAGAGATGGTTCCTTCAGTTGATATTGATAAAGATTATTTATATATAGAAAAACTGTCTCATGATAATTATTTTGCAAAGTTTGCACTAGAGTATGGGAAGGTGTTGTTCTGCCCTGAGCAACTGGTCTTGTATCGAAGACACGGACATAATGTAACAACTAGTCATCATTTTAAATTATCTCCGCTAAA

>CQ2616-Oct2011

GGGGGTTTTAATGGCAGGTTTTTATCATGCATTGCTAGAGATGGTTCCTTCAGTTGATATTGATAAAGATTATTTATATATAGAAAAACTGGCTCATGATAGTTATTTTGCAAAGTTTGCACTAGAGTATGGGAAGGTGTTGTTCTGCCCTGAGCAACTGGTCTTGTATCGAAGACATGGACATAATGTAACAACTAGTCATCATTTTAAATTATCTCCGCTAA

>CQ4048-Dec2012

GGCCCCCTTCCTTCTCATTTTTAATTATTTGCGCTAGATGGTTCCTTCAGTTGATATTGATAAAGATTATTTATATATAGAAAAACTGGCTCATGATAGTTATTTTGCAAAGTTTGCACTAGAGTATGGGAAGGTGTTGTTCTGCCCTGAGCAACTGGTCTTGTATCGAAGACATGGACATAATGTAACAACTAGTCATCATTTTAAATTATCTCCGCTAAA

>CQ4082-Dec2012

GGCCCCCTTCCTTCTCATTTTTAATTATTTGCGCTAGATGGTTCCTTCAGTTGATATTGATAAAGATTATTTATATATAGAAAAACTGGCTCATGATAGTTATTTTGCAAAGTTTGCACTAGAGTATGGGAAGGTGTTGTTCTGCCCTGAGCAACTGGTCTTGTATCGAAGACATGGACATAATGTAACAACTAGTCATCATTTTAAATTATCTCCGCTAAA

>CQ4158-Dec2013

GGGGGGGGGTCGGGAGGCTTTATCATGCATTGTTAGAGATGGTTCCTTCAGTTGATATTGATAAAGATTATTTATATATAGAAAAACTGTCTCATGATAATTATTTTGCAAAGTTTGCACTAGAGTATGGGAAGGTGTTGTTCTGCCCTGAGCAACTGGTCTTGTATCGAAGACACGGACATAATGTAACAACTAGTCATCATTTTAAATTATCTCCGCTAAA

>CQ4166-Dec2013

GGGGGGGTGTCGGGGCAGGCTTTATCATGCATTGTTAGAGATGGTTCCTTCAGTTGATATTGATAAAGATTATTTATATATAGAAAAACTGTCTCATGATAATTATTTTGCAAAGTTTGCACTAGAGTATGGGAAGGTGTTGTTCTGCCCTGAGCAACTGGTCTTGTATCGAAGACACGGACATAATGTAACAACTAGTCATCATTTTAAATTATCTCCGCTAAA

>CQ4589-Mar2013

GGGGGGTTTTCATGCGGTTTTAATCATGCATTGCTAGAGATGGTTCCTTCAGTTGATATTGATAAAGATTATTTATATATAGAAAAACTGGCTCATGATAGTTATTTTGCAAAGTTTGCGCTAGAGTATGGGAAGGTGTTGTTCTGCCCTGAACAACTGGTCTTGTATCGAAGACATGGACATAATGTAACAACTAGTCATCATTTTAAATTATCTCCGCTAA

>CQ4961-Jun2013

GTGAATGTCTTATGTCATGTCTTCGATACAAGACCAGTTGCTCAGGGCAGAACAACACCTTCCCATACTCTAGTGCAAACTTTGCAAAATAACTATCATGAGCCAGTTTTTCTATATATAAATAATCTTTATCAATATCAACTGAAGGAACCATCTCTAGCAATGCATGATTAAAACCTGCAGTACACCCCCAGATATAGGCATGAATAAATACAAATTTGA

>CQ5495-Oct2013

GAGGTTGGGTACATATAGTGCGATTTCTCATGCATTGCTAGAGATGGTTCCTTCAGTTGATATTGATAAAGATTATTTATATATAGAAAAACTGGCTCATGATAGTTATTTTGCAAAGTTTGCACTAGAGTATGGGAAGGTGTTGTTCTGCCCTGAGCAACTGGTCTTGTATCGAAGACATGGACATAATGTAACAACTAGTCATCATTTTAAATTATCTCCGCTAAA

>CQ5690-Nov2013

GGGGGGGGTACCTGCCAGGTTTTATCATGCATTGCTAGAGATGGTTCCTTCAGTTGATATTGATAAAGATTATTTATATATAGAAAAACTGGCTCATGATAGTTATTTTGCAAAGTTTGCACTAGAGTATGGGAAGGTGTTGTTCTGCCCTGAGCAACTGGTCTTGTATCGAAGACATGGACATAATGTAACAACTAGTCATCATTTTAAATTATCTCCGCTAAA

>CQ6078-Jan2014

GGGGGGGGGTTCTGGCAGGTTTTATCATGCATTGCTAGAGATGGTTCCTTCAGTTGATATTGATAAAGATTATTTATATATAGAAAAACTGGCTCATGATAGTTATTTTGCAAAGTTTGCACTAGAGTATGGGAAGGTGTTGTTCTGCCCTGAGCAACTGGTCTTGTATCGAAGACATGGACATAATGTAACAACTAGTCATCATTTTAAATTATCTCCGCTAA

>CQ6733-Jun2014

GGGGGGGGGTTCTGGCAGGTTTTATCATGCATTGCTAGAGATGGTTCCTTCAGTTGATATTGATAAAGATTATTTATATATAGAAAAACTGGCTCATGATAGTTATTTTGCAAAGTTTGCACTAGAGTATGGGAAGGTGTTGTTCTGCCCTGAGCAACTGGTCTTGTATCGAAGACATGGACATAATGTAACAACTAGTCATCATTTTAAATTATCTCCGCTAA

>CQ2854-Jur2012

GGGGGTTTTAATGGCAGGTTTTTATCATGCATTGCTAGAGATGGTTCCTTCAGTTGATATTGATAAAGATTATTTATATATAGAAAAACTGGCTCATGATAGTTATTTTGCAAAGTTTGCACTAGAGTATGGGAAGGTGTTGTTCTGCCCTGAGCAACTGGTCTTGTATCGAAGACATGGACATAATGTAACAACTAGTCATCATTTTAAATTATCTCCGCTAA

>CQ7967-Feb2015

GGGGGGGTCGGCAGGCTTTAATCATGCATTGTTAGAGATGGTTCCTTCAGTTGATATTGATAAAGATTATTTATATATAGAAAAACTGTCTCATGATAATTATTTTGCAAAGTTTGCACTAGAGTATGGGAAGGTGTTGTTCTGCCCTGAGCAACTGGTCTTGTATCGAAGACACGGACATAATGTAACAACTAGTCATCATTTTAAATTATCTCCGCTAAA

>CQ7565-Nov2014

CCGAATGATATTATGTCATGTCTTCGATACAAGACCAGTTGCTCAGGGCAGAACAACACCTTCCCATACTCTAGTGCAAACTTTGCAAAATAACTATCATGAGCCAGTTTTTCTATATATAAATAATCTTTATCAATATCAACTGAAGGAACCATCTCTAGCAATGCATGATTAAAACCTGCAGTACACCCCCAGATATAGGCATGAATAAAATACAAATTAACA

>CQ8479-Jul2015

AGGTTGTTAATTAGGAGGCTTTATCATGCATTGTTAGAGATGGTTCCTTCAGTTGATATTGATAAAGATTATTTATATATAGAAAAACTGTCTCATGATAATTATTTTGCAAAGTTTGCACTAGAGTATGGGAAGGTGTTGTTCTGCCCTGAGCAACTGGTCTTGTATCGAAGACACGGACATAATGTAACAACTAGTCATCATTTTAAATTATCTCCGCTAAA

>

**15B/C**

>CQ1504-Mar2011

TATCAGTGTTGTTTTAGATTTGAGTAAGGCATTCAATTGGATATTAGCAGTATTTTTTTATAATTATTATTTGAAAAATCCCATTAACGTTGACAAGATAAAGAAATATATGTTTTATAATTTCGCTATATTAGTTATTATTGTTGCTTTATTCTATGTTCAAAGAGGCGCTAATGTAGTATTGTTTGGAAGAAGCTTATTAGGTTGGGACGGATTCGTATCAGCTACCAGTTACGGAGTAAGATATGCAGGATTTTTAGAATATTCAACATTAAATGGGCAGTTGATTCTTTTTTTGTTACCGTTAATTAGGTTATTTAAACTTAGTTTTTTTACACAAGTAACTATTCTTGCTTTTTTGCTACAGGTTTTAGTATTGAGTAAATCTAGAATAGCTATTATTGCTCTGATTATATACATAGTATTTGTAGTAATGGTTCAGATTACTTCAATTATAAAGCGGATGA

>CQ1510-Mar2011

AATCGTGATGTTTTAGATTTGAGTAAGGCATTCAATTGGATATTAGCAGTATTTTTTTATAATTATTATTTGAAAAATCCCATTAACGTTGACAAGATAAAGAAATATATGTTTTATAATTTCGCTATATTAGTTATTATTGTTGCTTTATTCTATGTTCAAAGAGGCGCTAATGTAGTATTGTTTGGAAGAAGCTTATTAGGTTGGGACGGATTCGTATCAGCTACCAGTTACGGAGTAAGATATGCAGGATTTTTAGAATATTCAACATTAAATGGGCAGTTGATTCTTTTTTTGTTACCGTTAATTAGGTTATTTAAACTTAGTTTTTTTACACAAGTAACTATTCTTGCTTTTTTGCTACAGGTTTTAGTATTGAGTAAATCTAGAATAGCTATTATTGCTCTGATTATATACATAGTATTTGTAGTAATGGTTCAGATTACTTCAATTATAAAAGCGGATGA

>CQ1532-Mar2011

TGGTGTTTTGTTTTCCGATTTGAGTGAGGCATTCAATTGGATATTAGCAGTATTTTTTTATAATTATTATTTGAAAAATCCCATTAACGTTGACAAGATAAAGAAATATATGTTTTATAATTTCGCTATATTAGTTATTATTGTTGCTTTATTCTATGTTCAAAGAGGCGCTAATGTAGTATTGTTTGGAAGAAGCTTATTAGGTTGGGACGGATTCGTATCAGCTACCAGTTACGGAGTAAGATATGCAGGATTTTTAGAATATTCAACATTAAATGGGCAGTTGATTCTTTTTTTGTTACCGTTAATTAGGTTATTTAAACTTAGTTTTTTTACACAAGTAACTATTCTTGCTTTTTTGCTACAGGTTTTAGTATTGAGTAAATCTAGAATAGCTATTATTGCTCTGATTATATACATAGTATTTGTAGTAATGGTTCAGATTACTTCAATTAATAAGCGGATGA

>CQ1926-Jul2011

TATTCGCTGTTGTTTTAGATTTGAGTAAGGCATTCAATTGGATATTAGCAGTATTTTTTTATAATTATTATTTGAAAAATCCCATTAACGTTGACAAGATAAAGAAATATATGTTTTATAATTTCGCTATATTAGTTATTATTGTTGCTTTATTCTATGTTCAAAGAGGCGCTAATGTAGTATTGTTTGGAAGAAGCTTATTAGGTTGGGACGGATTCGTATCAGCTACCAGTTACGGAGTAAGATATGCAGGATTTTTAGAATATTCAACATTAAATGGGCAGTTGATTCTTTTTTTGTTACCGTTAATTAGGTTATTTAAACTTAGTTTTTTTACACAAGTAACTATTCTTGCTTTTTTGCTACAGGTTTTAGTATTGAGTAAATCTAGAATAGCTATTATTGCTCTGATTATATACATAGTATTTGTAGTAATGGTTCAGATTACTTCAATTATAAAAGCGGATGA

>CQ2395-Nov2011

AATACGTGTTGTTTTAGATTTGAGTAAGGCATTCAATTGGATATTAGCAGTATTTTTTTATAATTATTATTTGAAAAATCCCATTAACGTTGACAAGATAAAGAAATATATGTTTTATAATTTCGCTATATTAGTTATTATTGTTGCTTTATTCTATGTTCAAAGAGGCGCTAATGTAGTATTGTTTGGAAGAAGCTTATTAGGTTGGGACGGATTCGTATCAGCTACCAGTTACGGAGTAAGATATGCAGGATTTTTAGAATATTCAACATTAAATGGGCAGTTGATTCTTTTTTTGTTACCGTTAATTAGGTTATTTAAACTTAGTTTTTTTACACAAGTAACTATTCTTGCTTTTTTGCTACAGGTTTTAGTATTGAGTAAATCTAGAATAGCTATTATTGCTCTGATTATATACATAGTATTTGTAGTAATGGTTCAGATTACTTCAATTA

>CQ4640-Apr2013

AATAGGTGTTGTTTTCAGATTTGAGTAAGGCATTCAATTGGATATTAGCAGTATTTTTTTATAATTATTATTTGAAAAATCCCATTAACGTTGACAAGATAAAGAAATATATGTTTTATAATTTCGCTATATTAGTTATTATTGTTGCTTTATTCTATGTTCAAAGAGGCGCTAATGTAGTATTGTTTGGAAGAAGCTTATTAGGTTGGGACGGATTCGTATCAGCTACCAGTTACGGAGTAAGATATGCAGGATTTTTAGAATATTCAACATTAAATGGGCAGTTGATTCTTTTTTTGTTACCGTTAATTAGGTTATTTAAACTTAGTTTTTTTACACAAGTAACTATTCTTGCTTTTTTGCTACAGGTTTTAGTATTGAGTAAATCTAGAATAGCTATTATTGCTCTGATTATATACATAGTATTTGTAGTAATGGTTCAGATTACTTCAATTATAAAAGCGGATGA

>CQ6794-Jun2014

AATAAGCTGTTGTTTTAGATTTGAGTAAGGCATTCAATTGGATATTAGCAGTATTTTTTTATAATTATTATTTGAAAAATCCCATTAACGTTGACAAGATAAAGAAATATATGTTTTATAATTTCGCTATATTAGTTATTATTGTTGCTTTATTCTATGTTCAAAGAGGCGCTAATGTAGTATTGTTTGGAAGAAGCTTATTAGGTTGGGACGGATTCGTATCAGCTACCAGTTACGGAGTAAGATATGCAGGATTTTTAGAATATTCAACATTAAATGGGCAGTTGATTCTTTTTTTGTTACCGTTAATTAGGTTATTTAAACTTAGTTTTTTTACACAAGTAACTATTCTTGCTTTTTTGCTACAGGTTTTAGTATTGAGTAAATCTAGAATAGCTATTATTGCTCTGATTATATACATAGTATTTGTAGTAATGGTTCAGATTACTTCAATTATAAAAGCGGATGA

>CQ7460-Nov2014

ATCAGGTGTTGTTTTAGATTTGAGTAAGGCATTCAATTGGATATTAGCAGTATTTTTTTATAATTATTATTTGAAAAATCCCATTAACGTTGACAAGATAAAGAAATATATGTTTTATAATTTCGCTATATTAGTTATTATTGTTGCTTTATTCTATGTTCAAAGAGGCGCTAATGTAGTATTGTTTGGAAGAAGCTTATTAGGTTGGGACGGATTCGTATCAGCTACCAGTTACGGAGTAAGATATGCAGGATTTTTAGAATATTCAACATTAAATGGGCAGTTGATTCTTTTTTTGTTACCGTTAATTAGGTTATTTAAACTTAGTTTTTTTACACAAGTAACTATTCTTGCTTTTTTGCTACAGGTTTTAGTATTGAGTAAATCTAGAATAGCTATTATTGCTCTGATTATATACATAGTATTTGTAGTAATGGTTCAGATTACTTCAATTATAAAAGCGGATGA

>CQ8848-Dec2015

GGGGTAATATTCTTCATTCTTCGGCTAATGCATTCAATTGGATATTAGCAGTATTTTTTTATAATTATTATTTGAAAAATCCCATTAACGTTGACAAGATAAAGAAATATATGTTTTATAATTTCGCTATATTAGTTATTATTGTTGCTTTATTCTATGTTCAAAGAGGCGCTAATGTAGTATTGTTTGGAAGAAGCTTATTAGGTTGGGACGGATTCGTATCAGCTACCAGTTACGGAGTAAGATATGCAGGATTTTTAGAATATTCAACATTAAATGGGCAGTTGATTCTTTTTTTGTTACCGTTAATTAGGTTATTTAAACTTAGTTTTTTTACACAAGTAACTATTCTTGCTTTTTTGCTACAGGTTTTAGTATTGAGTAAATCTAGAATAGCTATTATTGCTCTGATTATATACATAGTATTTGTAGTAATGGTTCAGATTACTTCAATTATAAAGGCGGATG

>CQ8852-Dec2015

AATAAAACAATACAATGTATATAATCAGAGCAATAATAGCTATTCTAGATTTACTCAATACTAAAACCTGTAGCAAAAAAGCAAGAATAGTTACTTGTGTAAAAAAACTAAGTTTAAATAACCTAATTAACGGTAACAAAAAAAGAATCAACTGCCCATTTAATGTTGAATATTCTAAAAATCCTGCATATCTTACTCCGTAACTGGTAGCTGATACGAATCCGTCCCAACCTAATAAGCTTCTTCCAAACAATACTACATTAGCGCCTCTTTGAACATAGAATAAAGCAACAATAATAACTAATATAGCGAAATTATAAAACATATATTTCTTTATCTTGTCAACGTTAATGGGATTTTTCAAATAATAATTATAAAAAAATACTGCTAATATCCAATTGAATGCCTTACTCAAATCTGAAAACAACACTTGAATAGGTAAGCCACTAATTAAAAAATTCCAAA

>CQ9306-Apr2016

AAAAAGTGATGTTTTAGATTTGAGTAGGCATTCAATTGGATATTAGCAGTATTTTTTTATAATTATTATTTGAAAAATCCCATTAACGTTGACAAGATAAAGAAATATATGTTTTATAATTTCGCTATATTAGTTATTATTGTTGCTTTATTCTATGTTCAAAGAGGCGCTAATGTAGTATTGTTTGGAAGAAGCTTATTAGGTTGGGACGGATTCGTATCAGCTACCAGTTACGGAGTAAGATATGCAGGATTTTTAGAATATTCAACATTAAATGGGCAGTTGATTCTTTTTTTGTTACCGTTAATTAGGTTATTTAAACTTAGTTTTTTTACACAAGTAACTATTCTTGCTTTTTTGCTACAGGTTTTAGTATTGAGTAAATCTAGAATAGCTATTATTGCTCTGATTATATACATAGTATTTGTAGTAATGGTTCAGATTACTTCAATTATAAAAAGCGGATGA

>CQ7630-Dec2014

ATCAGCGTTGTTTTAGATTTGAGTAAGGCATTCAATTGGATATTAGCAGTATTTTTTTATAATTATTATTTGAAAAATCCCATTAACGTTGACAAGATAAAGAAATATATGTTTTATAATTTCGCTATATTAGTTATTATTGTTGCTTTATTCTATGTTCAAAGAGGCGCTAATGTAGTATTGTTTGGAAGAAGCTTATTAGGTTGGGACGGATTCGTATCAGCTACCAGTTACGGAGTAAGATATGCAGGATTTTTAGAATATTCAACATTAAATGGGCAGTTGATTCTTTTTTTGTTACCGTTAATTAGGTTATTTAAACTTAGTTTTTTTACACAAGTAACTATTCTTGCTTTTTTGCTACAGGTTTTAGTATTGAGTAAATCTAGAATAGCTATTATTGCTCTGATTATATACATAGTATTTGTAGTAATGGTTCAGATTACTTCAATTA

>CQ2274-Oct2011

AATCCGTGTTGATTTAGATTTGAGTAAGGCATTCAATTGGATATTAGCAGTATTTTTTTATAATTATTATTTGAAAAATCCCATTAACGTTGACAAGATAAAGAAATATATGTTTTATAATTTCGCTATATTAGTTATTATTGTTGCTTTATTCTATGTTCAAAGAGGCGCTAATGTAGTATTGTTTGGAAGAAGCTTATTAGGTTGGGACGGATTCGTATCAGCTACCAGTTACGGAGTAAGATATGCAGGATTTTTAGAATATTCAACATTAAATGGGCAGTTGATTCTTTTTTTGTTACCGTTAATTAGGTTATTTAAACTTAGTTTTTTTACACAAGTAACTATTCTTGCTTTTTTGCTACAGGTTTTAGTATTGAGTAAATCTAGAATAGCTATTATTGCTCTGATTATATACATAGTATTTGTAGTAATGGTTCAGATTACTTCAATTAAAAAAGCGGATGA

>CQ6738-Jun2014

TAGAGTTTGTTTTAGATTTGAGTAAGGCATTCAATTGGATATTAGCAGTATTTTTTTATAATTATTATTTGAAAAATCCCATTAACGTTGACAAGATAAAGAAATATATGTTTTATAATTTCGCTATATTAGTTATTATTGTTGCTTTATTCTATGTTCAAAGAGGCGCTAATGTAGTATTGTTTGGAAGAAGCTTATTAGGTTGGGACGGATTCGTATCAGCTACCAGTTACGGAGTAAGATATGCAGGATTTTTAGAATATTCAACATTAAATGGGCAGTTGATTCTTTTTTTGTTACCGTTAATTAGGTTATTTAAACTTAGTTTTTTTACACAAGTAACTATTCTTGCTTTTTTGCTACAGGTTTTAGTATTGAGTAAATCTAGAATAGCTATTATTGCTCTGATTATATACATAGTATTTGTAGTAATGGTTCAGATTACTTCAATTATAAAAGCGGAGGA

>CQ5301-Aug2013

AGATCCGTGATGTTTTAGATTTGAGTAAGGCATTCAATTGGATATTAGCAGTATTTTTTTATAATTATTATTTGAAAAATCCCATTAACGTTGACAAGATAAAGAAATATATGTTTTATAATTTCGCTATATTAGTTATTATTGTTGCTTTATTCTATGTTCAAAGAGGCGCTAATGTAGTATTGTTTGGAAGAAGCTTATTAGGTTGGGACGGATTCGTATCAGCTACCAGTTACGGAGTAAGATATGCAGGATTTTTAGAATATTCAACATTAAATGGGCAGTTGATTCTTTTTTTGTTACCGTTAATTAGGTTATTTAAACTTAGTTTTTTTACACAAGTAACTATTCTTGCTTTTTTGCTACAGGTTTTAGTATTGAGTAAATCTAGAATAGCTATTATTGCTCTGATTATATACATAGTATTTGTAGTAATGGTTCAGATTACTTCAATTA

>CQ5399-Sep2013

AGATCCGTGATGTTTTAGATTTGAGTAAGGCATTCAATTGGATATTAGCAGTATTTTTTTATAATTATTATTTGAAAAATCCCATTAACGTTGACAAGATAAAGAAATATATGTTTTATAATTTCGCTATATTAGTTATTATTGTTGCTTTATTCTATGTTCAAAGAGGCGCTAATGTAGTATTGTTTGGAAGAAGCTTATTAGGTTGGGACGGATTCGTATCAGCTACCAGTTACGGAGTAAGATATGCAGGATTTTTAGAATATTCAACATTAAATGGGCAGTTGATTCTTTTTTTGTTACCGTTAATTAGGTTATTTAAACTTAGTTTTTTTACACAAGTAACTATTCTTGCTTTTTTGCTACAGGTTTTAGTATTGAGTAAATCTAGAATAGCTATTATTGCTCTGATTATATACATAGTATTTGTAGTAATGGTTCAGATTACTTCAATTA

>CQ7292-Nov2014

ATCAGGTGTTGTTTTAGATTTGAGTAAGGCATTCAATTGGATATTAGCAGTATTTTTTTATAATTATTATTTGAAAAATCCCATTAACGTTGACAAGATAAAGAAATATATGTTTTATAATTTCGCTATATTAGTTATTATTGTTGCTTTATTCTATGTTCAAAGAGGCGCTAATGTAGTATTGTTTGGAAGAAGCTTATTAGGTTGGGACGGATTCGTATCAGCTACCAGTTACGGAGTAAGATATGCAGGATTTTTAGAATATTCAACATTAAATGGGCAGTTGATTCTTTTTTTGTTACCGTTAATTAGGTTATTTAAACTTAGTTTTTTTACACAAGTAACTATTCTTGCTTTTTTGCTACAGGTTTTAGTATTGAGTAAATCTAGAATAGCTATTATTGCTCTGATTATATACATAGTATTTGTAGTAATGGTTCAGATTACTTCAATTATAAAAGCGGATGA

>CQ3420-May2012

AGGGCCTCTGATCGTTTTAGATTTGAGTAAGCATTCAATTGGATATTAGCAGTATTTTTTTATAATTATTATTTGAAAAATCCCATTAACGTTGACAAGATAAAGAAATATATGTTTTATAATTTCGCTATATTAGTTATTATTGTTGCTTTATTCTATGTTCAAAGAGGCGCTAATGTAGTATTGTTTGGAAGAAGCTTATTAGGTTGGGACGGATTCGTATCAGCTACCAGTTACGGAGTAAGATATGCAGGATTTTTAGAATATTCAACATTAAATGGGCAGTTGATTCTTTTTTTGTTACCGTTAATTAGGTTATTTAAACTTAGTTTTTTTACACAAGTAACTATTCTTGCTTTTTTGCTACAGGTTTTAGTATTGAGTAAATCTAGAATAGCTATTATTGCTCTGATTATATACATAGTATTTGTAGTAATGGTTCAGATTACTTCAATTATAAGCGGATGA

**19A**

>CQ90-Jul2009

GCGTTCATCTATGAGTGTGTGTATAATCCTCTTGAACAGTAGTTGTAAGAGCATCAATGACAGTATTTCCAGTCACATAAACATTCTCTCTACCTTCTTTTAAGAGATTTTCTTTAGCCAACTCAGTTGGAGCAAAATGGTAAGTTGCAATGATTGATGTCGATTGCCTGTTAAATTCTTCAGGAAATGGACTTTGTAAATTGTACGTTCGCAAACCAGCTTCAACATGTCCTACTTTAATTCCCAAATAGAATGCTGCCAAGGCTGCTGCATAAGTTGTCGTAGTGTCACCGTGAACTAGGACAATATCTGGTTGTTCCTTCTCTAAAACTGGTTTTATCTTTTCCAAGATACTAGTTGTGATAGAGAACAAGGTTTGGTTAGCCTTCATAATTTCTAAATCATAATCTGGTACATCACCAAATAAATCTAAAAATGACTAAC

>CQ240-Nov2009

GAATTAGTCATGATTCAGAATTATAGAAGGCTTACCAAACCATGCATCTCTATCACAACTAGTATCTTGGAAAAGATAAAACCAGTTTTAGAGAAGGAACAACCAGATATTGTCCTAGTTCACGGTGACACTACGACAACTTATGCAGCAGCCTTGGCAGCATTCTATTTGGGAATTAAAGTAGGACATGTTGAAGCTGGTTTGCGAACGTACAATTTACAAAGTCCATTTCCTGAAGAATTTAACAGGCAATCGACATCAATCATTGCAACTTACCATTTTGCTCCAACTGAGTTGGCTAAAGAAAATCTCTTAAAAGAAGGTAGAGAGAATGTTTATGTGACTGGAAATACTGTCATTGATGCTCTTACAACTACTGTTCAAGAGGATTATACACACACTCATTTAGATTTAAACGCTAACTATCGTCTCATCTTTTGGGACTGCTCA

>CQ644-Jul2010

CTGTATACTGTCGTGTCTAGATGCTATAGATCAGCTTACCGATCCTTGCATCTCTATCACAACTAGTATCTTGGAAAAGATAAAACCAGTTTTAGAGAAGGAACAACCAGATATTGTCCTAGTTCACGGTGACACTACGACAACTTATGCAGCAGCCTTGGCAGCATTCTATTTGGGAATTAAAGTAGGACATGTTGAAGCTGGTTTGCGAACGTACAATTTACAAAGTCCATTTCCTGAAGAATTTAACAGGCAATCGACATCAATCATTGCAACTTACCATTTTGCTCCAACTGAGTTGGCTAAAGAAAATCTCTTAAAAGAAGGTAGAGAGAATGTTTATGTGACTGGAAATACTGTCATTGATGCTCTTACAACTACTGTTCAAGAGGATTATACACACACTCATTTAGATTTAAACGCTAACTATCGTCTCATCTTATTGACTGCTCA

>CQ3694-Jul2012

CGGCTTATGATTTAGATTATGAAGGCTAACCAAACCTTGTTCTCTATCACAACTAGTATCTTGGAAAAGATAAAACCAGTTTTAGAGAAGGAACAACCAGATATTGTCCTAGTTCACGGTGACACTACGACAACTTATGCAGCAGCCTTGGCAGCATTCTATTTGGGAATTAAAGTAGGACATGTTGAAGCTGGTTTGCGAACGTACAATTTACAAAGTCCATTTCCTGAAGAATTTAACAGGCAATCGACATCAATCATTGCAACTTACCATTTTGCTCCAACTGAGTTGGCTAAAGAAAATCTCTTAAAAGAAGGTAGAGAGAATGTTTATGTGACTGGAAATACTGTCATTGATGCTCTTACAACTACTGTTCAAGAGGATTATACACACACTCATTTAGATTTAAACGCTAACTATCGTCTCATCTTTTGACTGCTCCAAA

>CQ4348-Jan2013

CAAGTATTTATGATTTTAGAATTATGATGGCTACCAAACCTATGTTCTCTATCACAACTAGTATCTTGGAAAAGATAAAACCAGTTTTAGAGAAGGAACAACCAGATATTGTCCTAGTTCACGGTGACACTACGACAACTTATGCAGCAGCCTTGGCAGCATTCTATTTGGGAATTAAAGTAGGACATGTTGAAGCTGGTTTGCGAACGTACAATTTACAAAGTCCATTTCCTGAAGAATTTAACAGGCAATCGACATCAATCATTGCAACTTACCATTTTGCTCCAACTGAGTTGGCTAAAGAAAATCTCTTAAAAGAAGGTAGAGAGAATGTTTATGTGACTGGAAATACTGTCATTGATGCTCTTACAACTACTGTTCAAGAGGATTATACACACACTCATTTAGATTTAAACGCTAACTATCGTCTCATCTTTTGGGACTGCTCC

>CQ4466-Feb2013

CAAGTATTATCGATCTATCTTAATGAATGGCTCACCAAACCTTGTATCTCTATCACAACTAGTATCTTGGAAAAGATAAAACCAGTTTTAGAGAAGGAACAACCAGATATTGTCCTAGTTCACGGTGACACTACGACAACTTATGCAGCAGCCTTGGCAGCATTCTATTTGGGAATTAAAGTAGGACATGTTGAAGCTGGTTTGCGAACGTACAATTTACAAAGTCCATTTCCTGAAGAATTTAACAGGCAATCGACATCAATCATTGCAACTTACCATTTTGCTCCAACTGAGTTGGCTAAAGAAAATCTCTTAAAAGAAGGTAGAGAGAATGTTTATGTGACTGGAAATACTGTCATTGATGCTCTTACAACTACTGTTCAAGAGGATTATACACACACTCATTTAGATTTAAACGCTAACTATCGTCTCATCTTTTGGACCTGCTC

>CQ4759-Apr2013

AAGCATTATCGATCTTAGCTTTATGATGGCTCACCAAACCTTGTATCTCTATCACAACTAGTATCTTGGAAAAGATAAAACCAGTTTTAGAGAAGGAACAACCAGATATTGTCCTAGTTCACGGTGACACTACGACAACTTATGCAGCAGCCTTGGCAGCATTCTATTTGGGAATTAAAGTAGGACATGTTGAAGCTGGTTTGCGAACGTACAATTTACAAAGTCCATTTCCTGAAGAATTTAACAGGCAATCGACATCAATCATTGCAACTTACCATTTTGCTCCAACTGAGTTGGCTAAAGAAAATCTCTTAAAAGAAGGTAGAGAGAATGTTTATGTGACTGGAAATACTGTCATTGATGCTCTTACAACTACTGTTCAAGAGGATTATACACACACTCATTTAGATTTAAACGCTAACTATCGTCTCATCTTTTGGAACTGCTCCA

>CQ6205-Feb2014

GCGATCACTCTAATGCTAAGAGTGTATAATACATCTTGAACAGTAGTTGTAAGAGCATCAATGACAGTATTTCCAGTCACATAAACATTCTCTCTACCTTCTTTTAAGAGATTTTCTTTAGCCAACTCAGTTGGAGCAAAATGGTAAGTTGCAATGATTGATGTCGATTGCCTGTTAAATTCTTCAGGAAATGGACTTTGTAAATTGTACGTTCGCAAACCAGCTTCAACATGTCCTACTTTAATTCCCAAATAGAATGCTGCCAAGGCTGCTGCATAAGTTGTCGTAGTGTCACCGTGAACTAGGACAATATCTGGTTGTTCCTTCTCTAAAACTGGTTTTATCTTTTCCAAGATACTAGTTGTGATAGAGAACAAGGTTTGGTTAGCCTTCATAATTTCTAAATCATAATCTGGTACATCACCAAATAAATCTAAAAAGGGACTAAC

>CQ6548-Apr2014

TCGGATTATGATTTAGAATTATGAAGGCTCACCAAACCTATGTTCTCTATCACAACTAGTATCTTGGAAAAGATAAAACCAGTTTTAGAGAAGGAACAACCAGATATTGTCCTAGTTCACGGTGACACTACGACAACTTATGCAGCAGCCTTGGCAGCATTCTATTTGGGAATTAAAGTAGGACATGTTGAAGCTGGTTTGCGAACGTACAATTTACAAAGTCCATTTCCTGAAGAATTTAACAGGCAATCGACATCAATCATTGCAACTTACCATTTTGCTCCAACTGAGTTGGCTAAAGAAAATCTCTTAAAAGAAGGTAGAGAGAATGTTTATGTGACTGGAAATACTGTCATTGATGCTCTTACAACTACTGTTCAAGAGGATTATACACACACTCATTTAGATTTAAACGCTAACTATCGTCTCATCT

>CQ7445-Nov2014

CGGTATTATGATTTAGAATTATGAAGGCTAACCAAACCTTGTTCTCTATCACAACTAGTATCTTGGAAAAGATAAAACCAGTTTTAGAGAAGGAACAACCAGATATTGTCCTAGTTCACGGTGACACTACGACAACTTATGCAGCAGCCTTGGCAGCATTCTATTTGGGAATTAAAGTAGGACATGTTGAAGCTGGTTTGCGAACGTACAATTTACAAAGTCCATTTCCTGAAGAATTTAACAGGCAATCGACATCAATCATTGCAACTTACCATTTTGCTCCAACTGAGTTGGCTAAAGAAAATCTCTTAAAAGAAGGTAGAGAGAATGTTTATGTGACTGGAAATACTGTCATTGATGCTCTTACAACTACTGTTCAAGAGGATTATACACACACTCATTTAGATTTAAACGCTAACTATCGTCTCATCTTTTGGAAACTGCTCCA

>CQ8498-Aug2015

AAACCGAATAATCGATTTAGCAATTATGAATGGCTCACCAAACCTATGTTCTCTATCACAACTAGTATCTTGGAAAAGATAAAACCAGTTTTAGAGAAGGAACAACCAGATATTGTCCTAGTTCACGGTGACACTACGACAACTTATGCAGCAGCCTTGGCAGCATTCTATTTGGGAATTAAAGTAGGACATGTTGAAGCTGGTTTGCGAACGTACAATTTACAAAGTCCATTTCCTGAAGAATTTAACAGGCAATCGACATCAATCATTGCAACTTACCATTTTGCTCCAACTGAGTTGGCTAAAGAAAATCTCTTAAAAGAAGGTAGAGAGAATGTTTATGTGACTGGAAATACTGTCATTGATGCTCTTACAACTACTGTTCAAGAGGATTATACACACACTCATTTAGATTTAAACGCTAACTATCGTCTCATCTTATTGGACTGCTCCA

>CQ9124-Feb2016

ACGTATTTATGATTTAGAATTATGAAGGCTAACCAAACCTTGTTCTCTATCACAACTAGTATCTTGGAAAAGATAAAACCAGTTTTAGAGAAGGAACAACCAGATATTGTCCTAGTTCACGGTGACACTACGACAACTTATGCAGCAGCCTTGGCAGCATTCTATTTGGGAATTAAAGTAGGACATGTTGAAGCTGGTTTGCGAACGTACAATTTACAAAGTCCATTTCCTGAAGAATTTAACAGGCAATCGACATCAATCATTGCAACTTACCATTTTGCTCCAACTGAGTTGGCTAAAGAAAATCTCTTAAAAGAAGGTAGAGAGAATGTTTATGTGACTGGAAATACTGTCATTGATGCTCTTACAACTACTGTTCAAGAGGATTATACACACACTCATTTAGATTTAAACGCTAACTATCGTCTCATCTTATGGGACTGCTCA

>CQ9224-Mar2016

CATGATTATGATTTAGAATTATGAAGGCTAACCAAACCTTGTTCTCTATCACAACTAGTATCTTGGAAAAGATAAAACCAGTTTTAGAGAAGGAACAACCAGATATTGTCCTAGTTCACGGTGACACTACGACAACTTATGCAGCAGCCTTGGCAGCATTCTATTTGGGAATTAAAGTAGGACATGTTGAAGCTGGTTTGCGAACGTACAATTTACAAAGTCCATTTCCTGAAGAATTTAACAGGCAATCGACATCAATCATTGCAACTTACCATTTTGCTCCAACTGAGTTGGCTAAAGAAAATCTCTTAAAAGAAGGTAGAGAGAATGTTTATGTGACTGGAAATACTGTCATTGATGCTCTTACAACTACTGTTCAAGAGGATTATACACACACTCATTTAGATTTAAACGCTAACTATCGTCTCATCTTATGGACTGCTCA

>CQ9380-May2016

AATTATTATGATTTAGATTATGAAGGCTAACCAAACCTTGTTCTCTATCACAACTAGTATCTTGGAAAAGATAAAACCAGTTTTAGAGAAGGAACAACCAGATATTGTCCTAGTTCACGGTGACACTACGACAACTTATGCAGCAGCCTTGGCAGCATTCTATTTGGGAATTAAAGTAGGACATGTTGAAGCTGGTTTGCGAACGTACAATTTACAAAGTCCATTTCCTGAAGAATTTAACAGGCAATCGACATCAATCATTGCAACTTACCATTTTGCTCCAACTGAGTTGGCTAAAGAAAATCTCTTAAAAGAAGGTAGAGAGAATGTTTATGTGACTGGAAATACTGTCATTGATGCTCTTACAACTACTGTTCAAGAGGATTATACACACACTCATTTAGATTTAAACGCTAACTATCGTCTCATCTTTTGGAACTGCTCA

>CQ6110-Jan2014

CATTATTATGATTTAGAATTATGAAGGCTAACCAAACCTTGTTCTCTATCACAACTAGTATCTTGGAAAAGATAAAACCAGTTTTAGAGAAGGAACAACCAGATATTGTCCTAGTTCACGGTGACACTACGACAACTTATGCAGCAGCCTTGGCAGCATTCTATTTGGGAATTAAAGTAGGACATGTTGAAGCTGGTTTGCGAACGTACAATTTACAAAGTCCATTTCCTGAAGAATTTAACAGGCAATCGACATCAATCATTGCAACTTACCATTTTGCTCCAACTGAGTTGGCTAAAGAAAATCTCTTAAAAGAAGGTAGAGAGAATGTTTATGTGACTGGAAATACTGTCATTGATGCTCTTACAACTACTGTTCAAGAGGATTATACACACACTCATTTAGATTTAAACGCTAACTATCGTCTCATCTTATGGAACTGCTCA

>CQ6946-Jul2014

AGCTACATCGTCTATCTTATTCGACTGCTTGACTGCTCACACTCACTATCACACTAGTATCTTGGAAAAGATAAAACCAGTTTTAGAGAAGGAACAACCAGATATTGTCCTAGTTCACGGTGACACTACGACAACTTATGCAGCAGCCTTGGCAGCATTCTATTTGGGAATTAAAGTAGGACATGTTGAAGCTGGTTTGCGAACGTACAATTTACAAAGTCCATTTCCTGAAGAATTTAACAGGCAATCGACATCAATCATTGCAACTTACCATTTTGCTCCAACTGAGTTGGCTAAAGAAAATCTCTTAAAAGAAGGTAGAGAGAATGTTTATGTGACTGGAAATACTGTCATTGATGCTCTTACAACTACTGTTCAAGAGGATTATACACACACTCATTTAGATTTAAACGCTAACTATCGTCTCATCTTATTGACTGCTCA

>CQ6985-Jul2014

CAGTATTATGATTTAGAATTATGAAGGCTACCAAACCTTGTTCTCTATCACAACTAGTATCTTGGAAAAGATAAAACCAGTTTTAGAGAAGGAACAACCAGATATTGTCCTAGTTCACGGTGACACTACGACAACTTATGCAGCAGCCTTGGCAGCATTCTATTTGGGAATTAAAGTAGGACATGTTGAAGCTGGTTTGCGAACGTACAATTTACAAAGTCCATTTCCTGAAGAATTTAACAGGCAATCGACATCAATCATTGCAACTTACCATTTTGCTCCAACTGAGTTGGCTAAAGAAAATCTCTTAAAAGAAGGTAGAGAGAATGTTTATGTGACTGGAAATACTGTCATTGATGCTCTTACAACTACTGTTCAAGAGGATTATACACACACTCATTTAGATTTAAACGCTAACTATCGTCTCATCTTTTGGCCTGCTCA

>CQ8326-May2015

TGATTTAGAATTATGAAGGCTAACCAAACCTTGTTCTCTATCACAACTAGTATCTTGGAAAAGATAAAACCAGTTTTAGAGAAGGAACAACCAGATATTGTCCTAGTTCACGGTGACACTACGACAACTTATGCAGCAGCCTTGGCAGCATTCTATTTGGGAATTAAAGTAGGACATGTTGAAGCTGGTTTGCGAACGTACAATTTACAAAGTCCATTTCCTGAAGAATTTAACAGGCAATCGACATCAATCATTGCAACTTACCATTTTGCTCCAACTGAGTTGGCTAAAGAAAATCTCTTAAAAGAAGGTAGAGAGAATGTTTATGTGACTGGAAATACTGTCATTGATGCTCTTACAACTACTGTTCAAGAGGATTATACACACACTCATTTAGATTTAAACGCTAACTATCGTCTCATCTTATGACTGCTCAAA

>CQ3531-Jun2012

GCGATACCTCATAATGCTAAGACTGGACTAACCACTTGAACCAGTAGTTGTAAGAGCATCAATGACAGTATTTCCAGTCACATAAACATTCTCTCTACCTTCTTTTAAGAGATTTTCTTTAGCCAACTCAGTTGGAGCAAAATGGTAAGTTGCAATGATTGATGTCGATTGCCTGTTAAATTCTTCAGGAAATGGACTTTGTAAATTGTACGTTCGCAAACCAGCTTCAACATGTCCTACTTTAATTCCCAAATAGAATGCTGCCAAGGCTGCTGCATAAGTTGTCGTAGTGTCACCGTGAACTAGGACAATATCTGGTTGTTCCTTCTCTAAAACTGGTTTTATCTTTTCCAAGATACTAGTTGTGATAGAGAACAAGGTTTGGTTAGCCTTCATAATTTCTAAATCATAATCTGGTACATCACCAAATAAATCTAAAAAGGGGACTAAC

>CQ734-Jul2010

TCATGAGATCTGAGTGCAACTTAGGAAAAGTAAAACCAGTTTTAGAGAAGGAACAACCAGATATTGTCCTAGTTCACGGTGACACTACGACAACTTATGCAGCAGCCTTGGCAGCATTCTATTTGGGAATTAAAGTAGGACATGTTGAAGCTGGTTTGCGAACGTACAATTTACAAAGTCCATTTCCTGAAGAATTTAACAGGCAATCGACATCAATCATTGCAACTTACCATTTTGCTCCAACTGAGTTGGCTAAAGAAAATCTCTTAAAAGAAGGTAGAGAGAATGTTTATGTGACTGGAAATACTGTCATTGATGCTCTTACAACTACTGTTCAAGAGGATTATACACACACTCATTTAGATTTAAACGCTAACTATCGTCTCATCTTATTGACTGCT

>CQ4193-Dec2012

GGCCTACCATCGTCTATCTTATTCGACTGCTTAGACTGCTCAAACACACTATCACACCTAGTATCTTGGAAAAGATAAAACCAGTTTTAGAGAAGGAACAACCAGATATTGTCCTAGTTCACGGTGACACTACGACAACTTATGCAGCAGCCTTGGCAGCATTCTATTTGGGAATTAAAGTAGGACATGTTGAAGCTGGTTTGCGAACGTACAATTTACAAAGTCCATTTCCTGAAGAATTTAACAGGCAATCGACATCAATCATTGCAACTTACCATTTTGCTCCAACTGAGTTGGCTAAAGAAAATCTCTTAAAAGAAGGTAGAGAGAATGTTTATGTGACTGGAAATACTGTCATTGATGCTCTTACAACTACTGTTCAAGAGGATTATACACACACTCATTTAGATTTAAACGCTAACTATCGTCTCATCTTATTGACTGCTCA

>CQ6878-Jul2014

AGCTACATCGTCTATCTTATTCGACTGCTTGACTGCTCACACTCACTATCACACTAGTATCTTGGAAAAGATAAAACCAGTTTTAGAGAAGGAACAACCAGATATTGTCCTAGTTCACGGTGACACTACGACAACTTATGCAGCAGCCTTGGCAGCATTCTATTTGGGAATTAAAGTAGGACATGTTGAAGCTGGTTTGCGAACGTACAATTTACAAAGTCCATTTCCTGAAGAATTTAACAGGCAATCGACATCAATCATTGCAACTTACCATTTTGCTCCAACTGAGTTGGCTAAAGAAAATCTCTTAAAAGAAGGTAGAGAGAATGTTTATGTGACTGGAAATACTGTCATTGATGCTCTTACAACTACTGTTCAAGAGGATTATACACACACTCATTTAGATTTAAACGCTAACTATCGTCTCATCTTATTGACTGCTCA

>

**19F**

>CQ83-Jul2009

CATTTTTTTGGGAATTGCTCTACTATTATAATTGATCTCTTTATTTTGTATCTTTACAGGAAGGAAATATACAACCTTGTTCTTAGTAATGGATATACGGGGTCAAATATTCAGTGGTTTTTTAGAAATGCAACGAGTTATGAAGGTGAATTGACAGTGCGAACTTTTATTCGAGTTCTCATTCGTGTTATTGACGTATCTGCTTATATTTTTGGATATACTTTTATTAATAATTTTCTTATCTATCGCCATAAACGCCCTAAAGACATATTACA

>CQ198-Sep2009

CAGTTTTGGAATTGCTCTAACTATTATAATTGATCTCTTTATTTTGTATCTTTACAGGAAGGAAATATACAACCTTGTTCTTAGTAATGGATATACGGGGTCAAATATTCAGTGGTTTTTTAGAAATGCAACGAGTTATGAAGGTGAATTGACAGTGCGAACTTTTATTCGAGTTCTCATTCGTGTTATTGACGTATCTGCTTATATTTTTGGATATACTTTTATTAATAATTTTCTTATCTATCGCCATAAACGCCCTAAAGACATATTAC

>CQ291-Dec2009

TGGCCGTAACGCCCTAAGACATATGCGCCGTCTCCTCAACTATTACGTATCTTTACAGGAAGGAAATATACAACCTTGTTCTTAGTAATGGATATACGGGGTCAAATATTCAGTGGTTTTTTAGAAATGCAACGAGTTATGAAGGTGAATTGACAGTGCGAACTTTTATTCGAGTTCTCATTCGTGTTATTGACGTATCTGCTTATATTTTTGGATATACTTTTATTAATAATTTTCTTATCTATCGCCATAAACGCCCTAAAGACATATTAC

>CQ355-Feb2010

AAGTTCTTGGACTTGCTCTACTATTATAATTGATCTCTTTATTTTGTATCTTTACAGGAAGGAAATATACAACCTTGTTCTTAGTAATGGATATACGGGGTCAAATATTCAGTGGTTTTTTAGAAATGCAACGAGTTATGAAGGTGAATTGACAGTGCGAACTTTTATTCGAGTTCTCATTCGTGTTATTGACGTATCTGCTTATATTTTTGGATATACTTTTATTAATAATTTTCTTATCTATCGCCATAAACGCCCTAAAGACATATTACA

>CQ374-Feb2010

AAGTTTTTGGAATTGCTCTACTATTATAATTGATCTCTTTATTTTGTATCTTTACAGGAAGGAAATATACAACCTTGTTCTTAGTAATGGATATACGGGGTCAAATATTCAGTGGTTTTTTAGAAATGCAACGAGTTATGAAGGTGAATTGACAGTGCGAACTTTTATTCGAGTTCTCATTCGTGTTATTGACGTATCTGCTTATATTTTTGGATATACTTTTATTAATAATTTTCTTATCTATCGCCATAAACGCCCTAAAGACATATTAC

>CQ442-Mar2010

ATTTCTGGAAATTGCTCTAACTATTATAATTGATCTCTTTATTTTGTATCTTTACAGGAAGGAAATATACAACCTTGTTCTTAGTAATGGATATACGGGGTCAAATATTCAGTGGTTTTTTAGAAATGCAACGAGTTATGAAGGTGAATTGACAGTGCGAACTTTTATTCGAGTTCTCATTCGTGTTATTGACGTATCTGCTTATATTTTTGGATATACTTTTATTAATAATTTTCTTATCTATCGCCATAAACGCCCTAAAGACATATTAC

>CQ570-May2010

CAATTTTTTGGGAATTGCTCTACTATTATAATTGATCTCTTTATTTTGTATCTTTACAGGAAGGAAATATACAACCTTGTTCTTAGTAATGGATATACGGGGTCAAATATTCAGTGGTTTTTTAGAAATGCAACGAGTTATGAAGGTGAATTGACAGTGCGAACTTTTATTCGAGTTCTCATTCGTGTTATTGACGTATCTGCTTATATTTTTGGATATACTTTTATTAATAATTTTCTTATCTATCGCCATAAACGCCCTAAAGACATATTACA

>CQ609-Jun2010

GGGAATTGAATTATTCATTCATGTATCTCAACCAATATAAGCAGATACGTCAATAACACGAATGAGAACTCGAATAAAAGTTCGCACTGTCAATTCACCTTCCTAACTCGTTGCATTTCTAAAAAACCACTGAATATTTGACCCCGTATATCCATTACTAAGAACAAGGTTGTATATTTCCTTCCTGTAAAGATACAAAATAAAGAGATCAATTATAATAGTTAGAGCAATTTTCCAAAATTGGATATCAATTAATCGATCAGCAATCTTAAC

>CQ610-Jun2010

ACCATTTTTTTGGAAATTGCTCTAACTATTATAATTGATCTCTTTATTTTGTATCTTTACAGGAAGGAAATATACAACCTTGTTCTTAGTAATGGATATACGGGGTCAAATATTCAGTGGTTTTTTAGAAATGCAACGAGTTATGAAGGTGAATTGACAGTGCGAACTTTTATTCGAGTTCTCATTCGTGTTATTGACGTATCTGCTTATATTTTTGGATATACTTTTATTAATAATTTTCTTATCTATCGCCATAAACGCCCTAAAGACATATTAC

>CQ640-Jul2010

ACTTCTTCATAACTGCTCTAGAATTATAACATGATCTCTTATATTTTGTATCTTTACAGGAAGGAAATATACAACCTTGTTCTTAGTAATGGATATACGGGGTCAAATATTCAGTGGTTTTTTAGAAATGCAACGAGTTATGAAGGTGAATTGACAGTGCGAACTTTTATTCGAGTTCTCATTCGTGTTATTGACGTATCTGCTTATATTTTTGGATATACTTTTATTAATAATTTTCTTATCTATCGCCATAAACGCCCTAAAGGACATATTACA

>CQ728-Jul2010

TCGCAAATTATTAATAAAAGTATATCCAAAAATATAAGCAGATACGTCAATAACACGAATGAGAACTCGAATAAAAGTTCGCACTGTCAATTCACCTTCATAACTCGTTGCATTTCTAAAAAACCACTGAATATTTGACCCCGTATATCCATTACTAAGAACAAGGTTGTATATTTCCTTCCTGTAAAGATACAAAATAAAGAGATCAATTATAATAGTTAGAGCAATTTTCCAAAATTGGATATCAATTAATCGATCACAATCTTAACC

>CQ1087-Nov2010

AGTTTTTGGGAATTGCTCTAACTATTATAATTGATCTCTTTATTTTGTATCTTTACAGGAAGGAAATATACAACCTTGTTCTTAGTAATGGATATACGGGGTCAAATATTCAGTGGTTTTTTAGAAATGCAACGAGTTATGAAGGTGAATTGACAGTGCGAACTTTTATTCGAGTTCTCATTCGTGTTATTGACGTATCTGCTTATATTTTTGGATATACTTTTATTAATAATTTTCTTATCTATCGCCATAAACGCCCTAAAGACATATTAC

>CQ1237-Jan2011

AACTTTTTTTGGATTGCTCTACTATTATAATTGATCTCTTTATTTTGTATCTTTACAGGAAGGAAATATACAACCTTGTTCTTAGTAATGGATATACGGGGTCAAATATTCAGTGGTTTTTTAGAAATGCAACGAGTTATGAAGGTGAATTGACAGTGCGAACTTTTATTCGAGTTCTCATTCGTGTTATTGACGTATCTGCTTATATTTTTGGATATACTTTTATTAATAATTTTCTTATCTATCGCCATAAACGCCCTAAAGACATATTAC

>CQ2493-Nov2011

AATCCTTTGGCGTAGTTGCTCTACTATTATAATACGATCTCTATTATTTTGTATCTTTACAGGAAGGAAATATACAACCTTGTTCTTAGTAATGGATATACGGGGTCAAATATTCAGTGGTTTTTTAGAAATGCAACGAGTTATGAAGGTGAATTGACAGTGCGAACTTTTATTCGAGTTCTCATTCGTGTTATTGACGTATCTGCTTATATTTTTGGATATACTTTTATTAATAATTTTCTTATCTATCGCCATAAACGCCCTAAAGACATATTACA

>CQ2514-Nov2011

AACCTCTGGCAATTGCTCTAACTATTATAATTGATCTCTTTATTTTGTATCTTTACAGGAAGGAAATATACAACCTTGTTCTTAGTAATGGATATACGGGGTCAAATATTCAGTGGTTTTTTAGAAATGCAACGAGTTATGAAGGTGAATTGACAGTGCGAACTTTTATTCGAGTTCTCATTCGTGTTATTGACGTATCTGCTTATATTTTTGGATATACTTTTATTAATAATTTTCTTATCTATCGCCATAAACGCCCTAAAGACATATTAC

>CQ2544-Nov2011

AATTCCTGTAATTTGCTCCTAACTATTTTTAAATGATCTCTTTATTTTGTATCTTTACAGGAAGGAAATATACAACCTTGTTCTTAGTAATGGATATACGGGGTCAAATATTCAGTGGTTTTTTAGAAATGCAACGAGTTATGAAGGTGAATTGACAGTGCGAACTTTTATTCGAGTTCTCATTCGTGTTATTGACGTATCTGCTTATATTTTTGGATATACTTTTATTAATAATTTTCTTATCTATCGCCATAAACGCCCTAAGAC

>CQ2574-Dec2011

AACTTTTTGGGAAATTGCTCTAACTATTATAATTGATCTCTTTATTTTGTATCTTTACAGGAAGGAAATATACAACCTTGTTCTTAGTAATGGATATACGGGGTCAAATATTCAGTGGTTTTTTAGAAATGCAACGAGTTATGAAGGTGAATTGACAGTGCGAACTTTTATTCGAGTTCTCATTCGTGTTATTGACGTATCTGCTTATATTTTTGGATATACTTTTATTAATAATTTTCTTATCTATCGCCATAAACGCCCTAAAGACATATTACA

>CQ2862-Jan2012

AAGTTTTGGGAATTGCTCTACTATTATAATTGATCTCTTTATTTTGTATCTTTACAGGAAGGAAATATACAACCTTGTTCTTAGTAATGGATATACGGGGTCAAATATTCAGTGGTTTTTTAGAAATGCAACGAGTTATGAAGGTGAATTGACAGTGCGAACTTTTATTCGAGTTCTCATTCGTGTTATTGACGTATCTGCTTATATTTTTGGATATACTTTTATTAATAATTTTCTTATCTATCGCCATAAACGCCCTAAAGACATATTAC

>CQ2875-Jan2012

CATTTTTTGGGAATTGCTCTAACTATTATAATTGATCTCTTTATTTTGTATCTTTACAGGAAGGAAATATACAACCTTGTTCTTAGTAATGGATATACGGGGTCAAATATTCAGTGGTTTTTTAGAAATGCAACGAGTTATGAAGGTGAATTGACAGTGCGAACTTTTATTCGAGTTCTCATTCGTGTTATTGACGTATCTGCTTATATTTTTGGATATACTTTTATTAATAATTTTCTTATCTATCGCCATAAACGCCCTAAAGACATATTAC

>CQ3024-Feb2012

CAGGTATGCAATTGCTCTACTATTATAATTGATCTCTTTATTTTGTATCTTTACAGGAAGGAAATATACAACCTTGTTCTTAGTAATGGATATACGGGGTCAAATATTCAGTGGTTTTTTAGAAATGCAACGAGTTATGAAGGTGAATTGACAGTGCGAACTTTTATTCGAGTTCTCATTCGTGTTATTGACGTATCTGCTTATATTTTTGGATATACTTTTATTAATAATTTTCTTATCTATCGCCATAAACGCCCTAAAGACATATTACA

>CQ3194-Mar2012

CCGGTTAGAGTGCTCTACTATTATAATTAGATCTCTTATCTTTCCGTATCTTTCCAGGAAGGAAATATACAACCTTAGTTCTTAGTAATGGATATACGGGGTCAAATATTCAGTGGTTTTTTAGAAATGCAACGAGTTATGAAGGTGAATTGACAGTGCGAACTTTTATTCGAGTTCTCATTCGTGTTATTGACGTATCTGCTTATATTTTTGGATATACTTTTATTAATAATTTTCTTATCTATCGCCATAAACGCCCTAAAGACATATTACC

>CQ3401-May2012

AACTTTTTTGGAAATTGCTCTACTATTATAATTGATCTCTTTATTTTGTATCTTTACAGGAAGGAAATATACAACCTTGTTCTTAGTAATGGATATACGGGGTCAAATATTCAGTGGTTTTTTAGAAATGCAACGAGTTATGAAGGTGAATTGACAGTGCGAACTTTTATTCGAGTTCTCATTCGTGTTATTGACGTATCTGCTTATATTTTTGGATATACTTTTATTAATAATTTTCTTATCTATCGCCATAAACGCCCTAAAGACATATTAC

>CQ3473-May2012

AATTTTTGGCATTGCTCTACTATTATAATTGATCTCTTTATTTTGTATCTTTACAGGAAGGAAATATACAACCTTGTTCTTAGTAATGGATATACGGGGTCAAATATTCAGTGGTTTTTTAGAAATGCAACGAGTTATGAAGGTGAATTGACAGTGCGAACTTTTATTCGAGTTCTCATTCGTGTTATTGACGTATCTGCTTATATTTTTGGATATACTTTTATTAATAATTTTCTTATCTATCGCCATAAACGCCCTAAAGACATATTACA

>CQ3551-Jun2012

CAGTCACTGCAATTTGCTCTAACTATTATAATTGATCTCTTTATTTTGTATCTTTACAGGAAGGAAATATACAACCTTGTTCTTAGTAATGGATATACGGGGTCAAATATTCAGTGGTTTTTTAGAAATGCAACGAGTTATGAAGGTGAATTGACAGTGCGAACTTTTATTCGAGTTCTCATTCGTGTTATTGACGTATCTGCTTATATTTTTGGATATACTTTTATTAATAATTTTCTTATCTATCGCCATAAACGCCCTAAGGACATATTACCA

>CQ3548-Jun2012

ATCGTAAATTATTATAAAAGTATATCCAAAAATATAAGCAGATACGTCAATAACACGAATGAGAACTCGAATAAAAGTTCGCACTGTCAATTCACCTTCATAACTCGTTGCATTTCTAAAAAACCACTGAATATTTGACCCCGTATATCCATTACTAAGAACAAGGTTGTATATTTCCTTCCTGTAAAGATACAAAATAAAGAGATCAATTATAATAGTTAGAGCAATTTTCCAAAATTGGATATCAATTAATCGATCAGCAATCTTAAC

>CQ3608-Jun2012

ATCGCAAATTATTATAAAAGTATATCCAAAAATATAAGCAGATACGTCAATAACACGAATGAGAACTCGAATAAAAGTTCGCACTGTCAATTCACCTTCATAACTCGTTGCATTTCTAAAAAACCACTGAATATTTGACCCCGTATATCCATTACTAAGAACAAGGTTGTATATTTCCTTCCTGTAAAGATACAAAATAAAGAGATCAATTATAATAGTTAGAGCAATTTTCCAAAATTGGATATCAATTAATCGATCACAATCTTAACCAACT

>CQ3668-Jul2012

AATCTTTTTGGGAATTGCTCTACTATTATAATTGATCTCTTTATTTTGTATCTTTACAGGAAGGAAATATACAACCTTGTTCTTAGTAATGGATATACGGGGTCAAATATTCAGTGGTTTTTTAGAAATGCAACGAGTTATGAAGGTGAATTGACAGTGCGAACTTTTATTCGAGTTCTCATTCGTGTTATTGACGTATCTGCTTATATTTTTGGATATACTTTTATTAATAATTTTCTTATCTATCGCCATAAACGCCCTAAAGACATATTACA

>CQ3812-Aug2012

AATTTTTGGAATTGCTCTACTATTATAATTGATCTCTTTATTTTGTATCTTTACAGGAAGGAAATATACAACCTTGTTCTTAGTAATGGATATACGGGGTCAAATATTCAGTGGTTTTTTAGAAATGCAACGAGTTATGAAGGTGAATTGACAGTGCGAACTTTTATTCGAGTTCTCATTCGTGTTATTGACGTATCTGCTTATATTTTTGGATATACTTTTATTAATAATTTTCTTATCTATCGCCATAAACGCCCTAAAGACATATTAC

>CQ4047-Nov2012

AGTTTTGGGGAAATTGGCTCTAACTATTATAATTGATCTCTTTATTTTGTATCTTTACAGGAAGGAAATATACAACCTTGTTCTTAGTAATGGATATACGGGGTCAAATATTCAGTGGTTTTTTAGAAATGCAACGAGTTATGAAGGTGAATTGACAGTGCGAACTTTTATTCGAGTTCTCATTCGTGTTATTGACGTATCTGCTTATATTTTTGGATATACTTTTATTAATAATTTTCTTATCTATCGCCATAAACGCCCTAAGGACATATTAAT

>CQ4165-Dec2012

AACTTTATGGGGAATTGCTCTAACTATTATAATTGATCTCTTTATTTTGTATCTTTACAGGAAGGAAATATACAACCTTGTTCTTAGTAATGGATATACGGGGTCAAATATTCAGTGGTTTTTTAGAAATGCAACGAGTTATGAAGGTGAATTGACAGTGCGAACTTTTATTCGAGTTCTCATTCGTGTTATTGACGTATCTGCTTATATTTTTGGATATACTTTTATTAATAATTTTCTTATCTATCGCCATAAACGCCCTAAAGACATATTACA

>CQ4215-Jan2013

AGATTATATCGCGCTCTCTGATATTTAGCGTGCGTACACTCACATATTTTGTATCTTTACAGGAAGGAAATATACAACCTTGTTCTTAGTAATGGATATACGGGGTCAAATATTCAGTGGTTTTTTAGAAATGCAACGAGTTATGAAGGTGAATTGACAGTGCGAACTTTTATTCGAGTTCTCATTCGTGTTATTGACGTATCTGCTTATATTTTTGGATATACTTTTATTAATAATTTTCTTATCTATCGCCATAAACGCCCTAAAGACATATAAC

>CQ4416-Feb2013

AGGTTTTGGGAATTGCTCTACTATTATAATTGATCTCTTTATTTTGTATCTTTACAGGAAGGAAATATACAACCTTGTTCTTAGTAATGGATATACGGGGTCAAATATTCAGTGGTTTTTTAGAAATGCAACGAGTTATGAAGGTGAATTGACAGTGCGAACTTTTATTCGAGTTCTCATTCGTGTTATTGACGTATCTGCTTATATTTTTGGATATACTTTTATTAATAATTTTCTTATCTATCGCCATAAACGCCCTAAAGACATATTAC

>CQ5390-Sep2013

AAGTTTTGGATTGCTCTACTATTATAATTGATCTCTTTATTTTGTATCTTTACAGGAAGGAAATATACAACCTTGTTCTTAGTAATGGATATACGGGGTCAAATATTCAGTGGTTTTTTAGAAATGCAACGAGTTATGAAGGTGAATTGACAGTGCGAACTTTTATTCGAGTTCTCATTCGTGTTATTGACGTATCTGCTTATATTTTTGGATATACTTTTATTAATAATTTTCTTATCTATCGCCATAAACGCCCTAAAGACATATTAC

>CQ5675-Nov2013

AAGCTCTGGAATTGCTCTAACTATTATAATTGATCTCTTTATTTTGTATCTTTACAGGAAGGAAATATACAACCTTGTTCTTAGTAATGGATATACGGGGTCAAATATTCAGTGGTTTTTTAGAAATGCAACGAGTTATGAAGGTGAATTGACAGTGCGAACTTTTATTCGAGTTCTCATTCGTGTTATTGACGTATCTGCTTATATTTTTGGATATACTTTTATTAATAATTTTCTTATCTATCGCCATAAACGCCCTAAAGACATATTAC

>CQ5898-Dec2013

AACTTTTGGTATTGCTCTAACTATTATAATTGATCTCTTTATTTTGTATCTTTACAGGAAGGAAATATACAACCTTGTTCTTAGTAATGGATATACGGGGTCAAATATTCAGTGGTTTTTTAGAAATGCAACGAGTTATGAAGGTGAATTGACAGTGCGAACTTTTATTCGAGTTCTCATTCGTGTTATTGACGTATCTGCTTATATTTTTGGATATACTTTTATTAATAATTTTCTTATCTATCGCCATAAACGCCCTAAGGACATATTACA

>CQ8765-Nov2015

TAACGTAATTATTAATAAAAGTATATCCAAAAATATAAGCAGATACGTCAATAACACGAATGAGAACTCGAATAAAAGTTCGCACTGTCAATTCACCTTCATAACTCGTTGCATTTCTAAAAAACCACTGAATATTTGACCCCGTATATCCATTACTAAGAACAAGGTTGTATATTTCCTTCCTGTAAAGATACAAAATAAAGAGATCAATTATAATAGTTAGAGCAATTTTCCAAAATTGGATATCAATTAATCGATCACAATCTTAACC

>CQ6034-Jan2014

AGGTTTTGGAAATTGCTCTAACTATTATAATTGATCTCTTTATTTTGTATCTTTACAGGAAGGAAATATACAACCTTGTTCTTAGTAATGGATATACGGGGTCAAATATTCAGTGGTTTTTTAGAAATGCAACGAGTTATGAAGGTGAATTGACAGTGCGAACTTTTATTCGAGTTCTCATTCGTGTTATTGACGTATCTGCTTATATTTTTGGATATACTTTTATTAATAATTTTCTTATCTATCGCCATAAACGCCCTAAAGACATATTACA

>CQ6198-Feb2014

AAGTCTTTAGCCCTATTCGCTCTATCTATTATAATTACATCTCTTATATTTTGTATCTTTACAGGAAGGAAATATACAACCTTGTTCTTAGTAATGGATATACGGGGTCAAATATTCAGTGGTTTTTTAGAAATGCAACGAGTTATGAAGGTGAATTGACAGTGCGAACTTTTATTCGAGTTCTCATTCGTGTTATTGACGTATCTGCTTATATTTTTGGATATACTTTTATTAATAATTTTCTTATCTATCGCCATAAACGCCCTAAAGACATATTAC

>CQ6519-Apr2014

AATTTTTTGGAAATTGCTCTACTATTATAATTGATCTCTTTATTTTGTATCTTTACAGGAAGGAAATATACAACCTTGTTCTTAGTAATGGATATACGGGGTCAAATATTCAGTGGTTTTTTAGAAATGCAACGAGTTATGAAGGTGAATTGACAGTGCGAACTTTTATTCGAGTTCTCATTCGTGTTATTGACGTATCTGCTTATATTTTTGGATATACTTTTATTAATAATTTTCTTATCTATCGCCATAAACGCCCTAAAGGACATATTACC

>CQ6694-May2014

AGCTTTTGGAATTGCTCTAACTATTATAATTGATCTCTTTATTTTGTATCTTTACAGGAAGGAAATATACAACCTTGTTCTTAGTAATGGATATACGGGGTCAAATATTCAGTGGTTTTTTAGAAATGCAACGAGTTATGAAGGTGAATTGACAGTGCGAACTTTTATTCGAGTTCTCATTCGTGTTATTGACGTATCTGCTTATATTTTTGGATATACTTTTATTAATAATTTTCTTATCTATCGCCATAAACGCCCTAAAGACATATTACA

>CQ6800-Jun2014

AGTTTTTGGTAATTGCTCTAACTATTATAACATGATCTCTTTATTTTGTATCTTTACAGGAAGGAAATATACAACCTTGTTCTTAGTAATGGATATACGGGGTCAAATATTCAGTGGTTTTTTAGAAATGCAACGAGTTATGAAGGTGAATTGACAGTGCGAACTTTTATTCGAGTTCTCATTCGTGTTATTGACGTATCTGCTTATATTTTTGGATATACTTTTATTAATAATTTTCTTATCTATCGCCATAAACGCCCTAAGAACATATTACT

>CQ6865-Jul2014

CTTTTCTTTCACAATTCGGCCATCTACGCCCTAAACCCGTTTTAATTTATTTTGTATCTTTACAGGAAGGAAATATACAACCTTGTTCTTAGTAATGGATATACGGGGTCAAATATTCAGTGGTTTTTTAGAAATGCAACGAGTTATGAAGGTGAATTGACAGTGCGAACTTTTATTCGAGTTCTCATTCGTGTTATTGACGTATCTGCTTATATTTTTGGATATACTTTTATTAATAATTTTCTTATCTATCGCCATAAACGCCCTAAAGACATATTA

>CQ6933-Jul2014

AGTCATTTCGTGGTGCATCTACTATTATAATACGTTTTCCATTATTTTGTATCTTTACAGGAAGGAAATATACAACCTTGTTCTTAGTAATGGATATACGGGGTCAAATATTCAGTGGTTTTTTAGAAATGCAACGAGTTATGAAGGTGAATTGACAGTGCGAACTTTTATTCGAGTTCTCATTCGTGTTATTGACGTATCTGCTTATATTTTTGGATATACTTTTATTAATAATTTTCTTATCTATCGCCATAAACGCCCTAAAGACATATTACA

>CQ8237-Apr2015

AAGGTAGGAATTGCTCTACTATTATAATTGATCTCTTTATTTTGTATCTTTACAGGAAGGAAATATACAACCTTGTTCTTAGTAATGGATATACGGGGTCAAATATTCAGTGGTTTTTTAGAAATGCAACGAGTTATGAAGGTGAATTGACAGTGCGAACTTTTATTCGAGTTCTCATTCGTGTTATTGACGTATCTGCTTATATTTTTGGATATACTTTTATTAATAATTTTCTTATCTATCGCCATAAACGCCCTAAAGACATATTAC

>CQ8273-May2015

AAATAAATTGGGGGAAATTGCTCTAACTATTATAATTGATCTCTTTATTTTGTATCTTTACAGGAAGGAAATATACAACCTTGTTCTTAGTAATGGATATACGGGGTCAAATATTCAGTGGTTTTTTAGAAATGCAACGAGTTATGAAGGTGAATTGACAGTGCGAACTTTTATTCGAGTTCTCATTCGTGTTATTGACGTATCTGCTTATATTTTTGGATATACTTTTATTAATAATTTTCTTATCTATCGCCATAAACGCCCTAAGAACATATTACA

>CQ8336-May2015

AAGTTTTGGGACTTGCTCTACTATTATAATTGATCTCTTTATTTTGTATCTTTACAGGAAGGAAATATACAACCTTGTTCTTAGTAATGGATATACGGGGTCAAATATTCAGTGGTTTTTTAGAAATGCAACGAGTTATGAAGGTGAATTGACAGTGCGAACTTTTATTCGAGTTCTCATTCGTGTTATTGACGTATCTGCTTATATTTTTGGATATACTTTTATTAATAATTTTCTTATCTATCGCCATAAACGCCCTAAAGACATATTACA

>CQ8557-Sep2015

AATTTTGGAATTGCTCTACTATTATAATTGATCTCTTTATTTTGTATCTTTACAGGAAGGAAATATACAACCTTGTTCTTAGTAATGGATATACGGGGTCAAATATTCAGTGGTTTTTTAGAAATGCAACGAGTTATGAAGGTGAATTGACAGTGCGAACTTTTATTCGAGTTCTCATTCGTGTTATTGACGTATCTGCTTATATTTTTGGATATACTTTTATTAATAATTTTCTTATCTATCGCCATAAACGCCCTAAGACATATTACA

>CQ9346-Apr2016

AAGGTTTTGGAATTGCTCTAACTATTATAATTGATCTCTTTATTTTGTATCTTTACAGGAAGGAAATATACAACCTTGTTCTTAGTAATGGATATACGGGGTCAAATATTCAGTGGTTTTTTAGAAATGCAACGAGTTATGAAGGTGAATTGACAGTGCGAACTTTTATTCGAGTTCTCATTCGTGTTATTGACGTATCTGCTTATATTTTTGGATATACTTTTATTAATAATTTTCTTATCTATCGCCATAAACGCCCTAAAGACATATTAC

>CQ8758-Nov2015

AGGTTAGCAATTATTAGTTCAGGTATATCCATAGATATAAGCAGATACGTCAATAACACGAATGAGAACTCGAATAAAAGTTCGCACTGTCAATTCACCTTCATAACTCGTTGCATTTCTAAAAAACCACTGAATATTTGACCCCGTATATCCATTACTAAGAACAAGGTTGTATATTTCCTTCCTGTAAAGATACAAAATAAAGAGATCAATTATAATAGTTAGAGCAATTTTCCAAAATTGGATATCAATTAATCGATCACAAATCTTAACTGCA

>CQ8796-Nov2015

AAGCATTGGTAATTGCTCTAACTATTATAATTGATCTCTTTATTTTGTATCTTTACAGGAAGGAAATATACAACCTTGTTCTTAGTAATGGATATACGGGGTCAAATATTCAGTGGTTTTTTAGAAATGCAACGAGTTATGAAGGTGAATTGACAGTGCGAACTTTTATTCGAGTTCTCATTCGTGTTATTGACGTATCTGCTTATATTTTTGGATATACTTTTATTAATAATTTTCTTATCTATCGCCATAAACGCCCTAAAGGACATATTACA

>CQ6663-May2014

AGCTTTTGGAATTGCTCTAACTATTATAATTGATCTCTTTATTTTGTATCTTTACAGGAAGGAAATATACAACCTTGTTCTTAGTAATGGATATACGGGGTCAAATATTCAGTGGTTTTTTAGAAATGCAACGAGTTATGAAGGTGAATTGACAGTGCGAACTTTTATTCGAGTTCTCATTCGTGTTATTGACGTATCTGCTTATATTTTTGGATATACTTTTATTAATAATTTTCTTATCTATCGCCATAAACGCCCTAAAGACATATTACA

>CQ4562-Mar2013

GAGGTTTCGTCACCCTATCGCTCTCCTACCTATAACATACATCTCTATATATTTTGTATCTTTACAGGAAGGAAATATACAACCTTGTTCTTAGTAATGGATATACGGGGTCAAATATTCAGTGGTTTTTTAGAAATGCAACGAGTTATGAAGGTGAATTGACAGTGCGAACTTTTATTCGAGTTCTCATTCGTGTTATTGACGTATCTGCTTATATTTTTGGATATACTTTTATTAATAATTTTCTTATCTATCGCCATAAACGCCCTAAAGACATATTACAA

>CQ6010-Jan2014

AGCTTTGGTAATTGCTCTACTATTATAATTGATCTCTTTATTTTGTATCTTTACAGGAAGGAAATATACAACCTTGTTCTTAGTAATGGATATACGGGGTCAAATATTCAGTGGTTTTTTAGAAATGCAACGAGTTATGAAGGTGAATTGACAGTGCGAACTTTTATTCGAGTTCTCATTCGTGTTATTGACGTATCTGCTTATATTTTTGGATATACTTTTATTAATAATTTTCTTATCTATCGCCATAAACGCCCTAAGAACATATTACA

>CQ4309-Feb2013

AATTTTTTGGGGAAATTGCTCTAACTATTATAATTGATCTCTTTATTTTGTATCTTTACAGGAAGGAAATATACAACCTTGTTCTTAGTAATGGATATACGGGGTCAAATATTCAGTGGTTTTTTAGAAATGCAACGAGTTATGAAGGTGAATTGACAGTGCGAACTTTTATTCGAGTTCTCATTCGTGTTATTGACGTATCTGCTTATATTTTTGGATATACTTTTATTAATAATTTTCTTATCTATCGCCATAAACGCCCTAAAGACATATTAC

>CQ4778-Apr2013

AAGCTTTTGGCAATTGCTCTAACTATTATAATTGATCTCTTTATTTTGTATCTTTACAGGAAGGAAATATACAACCTTGTTCTTAGTAATGGATATACGGGGTCAAATATTCAGTGGTTTTTTAGAAATGCAACGAGTTATGAAGGTGAATTGACAGTGCGAACTTTTATTCGAGTTCTCATTCGTGTTATTGACGTATCTGCTTATATTTTTGGATATACTTTTATTAATAATTTTCTTATCTATCGCCATAAACGCCCTAAAGACATATTACA

>CQ5280-Aug2013

AAGCTTTGCAATTGCTCTAACTATTATAATTGATCTCTTTATTTTGTATCTTTACAGGAAGGAAATATACAACCTTGTTCTTAGTAATGGATATACGGGGTCAAATATTCAGTGGTTTTTTAGAAATGCAACGAGTTATGAAGGTGAATTGACAGTGCGAACTTTTATTCGAGTTCTCATTCGTGTTATTGACGTATCTGCTTATATTTTTGGATATACTTTTATTAATAATTTTCTTATCTATCGCCATAAACGCCCTAAAGAACATATTAC

>CQ7162-Sep2014

TTAGCAATTATTATAAAAGTATATCCAAAAATATAAGCAGATACGTCAATAACACGAATGAGAACTCGAATAAAAGTTCGCACTGTCAATTCACCTTCATAACTCGTTGCATTTCTAAAAAACCACTGAATATTTGACCCCGTATATCCATTACTAAGAACAAGGTTGTATATTTCCTTCCTGTAAAGATACAAAATAAAGAGATCAATTATAATAGTTAGAGCAATTTTCCAAAATTGGATATCAATTAATCGATCACAAATCTTAAATGAA

>CQ7382-Oct2014

AATCCTGTGATATCTGCTCTACGCCCTTAACATCGTTTTCTTTATTTTGTATCTTTACAGGAAGGAAATATACAACCTTGTTCTTAGTAATGGATATACGGGGTCAAATATTCAGTGGTTTTTTAGAAATGCAACGAGTTATGAAGGTGAATTGACAGTGCGAACTTTTATTCGAGTTCTCATTCGTGTTATTGACGTATCTGCTTATATTTTTGGATATACTTTTATTAATAATTTTCTTATCTATCGCCATAAACGCCCTAAAGGACATATTACA

>CQ8151-Apr2015

ATTTTGGGGAATTGCTCTAACTATTATAATTGATCTCTTTATTTTGTATCTTTACAGGAAGGAAATATACAACCTTGTTCTTAGTAATGGATATACGGGGTCAAATATTCAGTGGTTTTTTAGAAATGCAACGAGTTATGAAGGTGAATTGACAGTGCGAACTTTTATTCGAGTTCTCATTCGTGTTATTGACGTATCTGCTTATATTTTTGGATATACTTTTATTAATAATTTTCTTATCTATCGCCATAAACGCCCTAAAGACATATTAC

>CQ9405-May2016

GGTACGCATAACGGCCTACAGACATATTACCCGTTCTCCATACATTTTGTATCTTTACAGGAAGGAAATATACAACCTTGTTCTTAGTAATGGATATACGGGGTCAAATATTCAGTGGTTTTTTAGAAATGCAACGAGTTATGAAGGTGAATTGACAGTGCGAACTTTTATTCGAGTTCTCATTCGTGTTATTGACGTATCTGCTTATATTTTTGGATATACTTTTATTAATAATTTTCTTATCTATCGCCATAAACGCCCTAAAGACATATTA

>CQ5036-Jun2013

AATTTTTGGAAATTGCTCTAACTATTATAATTGATCTCTTTATTTTGTATCTTTACAGGAAGGAAATATACAACCTTGTTCTTAGTAATGGATATACGGGGTCAAATATTCAGTGGTTTTTTAGAAATGCAACGAGTTATGAAGGTGAATTGACAGTGCGAACTTTTATTCGAGTTCTCATTCGTGTTATTGACGTATCTGCTTATATTTTTGGATATACTTTTATTAATAATTTTCTTATCTATCGCCATAAACGCCCTAAAGACATATTACA

>CQ1056-Nov2011

TGTCGTAACGCCCTTAGACATATTCCCGGATCTCATTATTTTGTATCTTTACAGGAAGGAAATATACAACCTTGTTCTTAGTAATGGATATACGGGGTCAAATATTCAGTGGTTTTTTAGAAATGCAACGAGTTATGAAGGTGAATTGACAGTGCGAACTTTTATTCGAGTTCTCATTCGTGTTATTGACGTATCTGCTTATATTTTTGGATATACTTTTATTAATAATTTTCTTATCTATCGCCATAAACGCCCTAAAGACATATTAC

>CQ4515-Mar2013

ATCCTCTTTGGGAAATTGCTCTAACTATTATAATTGATCTCTTTATTTTGTATCTTTACAGGAAGGAAATATACAACCTTGTTCTTAGTAATGGATATACGGGGTCAAATATTCAGTGGTTTTTTAGAAATGCAACGAGTTATGAAGGTGAATTGACAGTGCGAACTTTTATTCGAGTTCTCATTCGTGTTATTGACGTATCTGCTTATATTTTTGGATATACTTTTATTAATAATTTTCTTATCTATCGCCATAAACGCCCTAAGAACATATTAC

>CQ5501-Oct2013

AAGTTTTTGGGGAATTGCTCTAACTATTATAATTGATCTCTATTATTTTGTATCTTTACAGGAAGGAAATATACAACCTTGTTCTTAGTAATGGATATACGGGGTCAAATATTCAGTGGTTTTTTAGAAATGCAACGAGTTATGAAGGTGAATTGACAGTGCGAACTTTTATTCGAGTTCTCATTCGTGTTATTGACGTATCTGCTTATATTTTTGGATATACTTTTATTAATAATTTTCTTATCTATCGCCATAAACGCCCTAAAGACATATTACA

>CQ1309-Jan2011

AGTCTGTATTGCTCTAACTATTATACATGATCTCTTTATTTTGTATCTTTACAGGAAGGAAATATACAACCTTGTTCTTAGTAATGGATATACGGGGTCAAATATTCAGTGGTTTTTTAGAAATGCAACGAGTTATGAAGGTGAATTGACAGTGCGAACTTTTATTCGAGTTCTCATTCGTGTTATTGACGTATCTGCTTATATTTTTGGATATACTTTTATTAATAATTTTCTTATCTATCGCCATAAACGCCCTAAAGACATATTAC

>CQ3432-May2012

AAGTATGGAATTTGCTCTAACTATTATAATTTGATCTCTTTTATTTTTGTATCTTTACAGGAAGGAAATATACAACCTTGTTCTTAGTAATGGATATACGGGGTCAAATATTCAGTGGTTTTTTAGAAATGCAACGAGTTATGAAGGTGAATTGACAGTGCGAACTTTTATTCGAGTTCTCATTCGTGTTATTGACGTATCTGCTTATATTTTTGGATATACTTTTATTAATAATTTTCTTATCTATCGCCATAAACGCCCTAAGAACATATTAAC

>CQ5487-Oct2013

AAGTTTTTGGGGAATTGCTCTAACTATTATAATTGATCTCTATTATTTTGTATCTTTACAGGAAGGAAATATACAACCTTGTTCTTAGTAATGGATATACGGGGTCAAATATTCAGTGGTTTTTTAGAAATGCAACGAGTTATGAAGGTGAATTGACAGTGCGAACTTTTATTCGAGTTCTCATTCGTGTTATTGACGTATCTGCTTATATTTTTGGATATACTTTTATTAATAATTTTCTTATCTATCGCCATAAACGCCCTAAAGACATATTACA

>CQ5685-Nov2013

ACTTTAGTTACGTAAACTGCATCTACGCCCTTAATACGTTTTAATTTATTTTGTATCTTTACAGGAAGGAAATATACAACCTTGTTCTTAGTAATGGATATACGGGGTCAAATATTCAGTGGTTTTTTAGAAATGCAACGAGTTATGAAGGTGAATTGACAGTGCGAACTTTTATTCGAGTTCTCATTCGTGTTATTGACGTATCTGCTTATATTTTTGGATATACTTTTATTAATAATTTTCTTATCTATCGCCATAAACGCCCTAAAGACATATTACA

**23F**

>CQ912-Sep2010

ACTATTATTATTATATGTTTGAAAAAATGTTAACGCCAGTAAAGAGATGACTATTGTCGTTGCTGTCACTACCAGTCTATGCTCTTTTTTAATATTATTTATAAAATTTCTATTAATAATAATCCAAAAAACAACTAGTATTATATAAACTGTTCGCGTATTAGATATTAAAAGTAAAAAAAGATCCAACCATAATACAAAACTATCAATCTGATGTTTAGCGTCATATTTTCTGCTGACATATAGTAGAATATAGGAAACTAAAATTGTAATTGCATAAAAATTTTTATGAGTCAAACCTCCATTAAAACCATATGAAAAGCCAATTCCCTCTACAGAAACTGTTACGTG

>CQ1696-May2011

TATAACTCGTACAATAGAGCTTTGAGTCATAGGTTTTGTATAGCAATTACAATTTTAGTTTCCTATATTCTACTATATGTCAGCAGAAAATATGACGCTAAACATCAGATTGATAGTTTTGTATTATGGTTAGATCTTTTTTTACTTTTAATATCTAATACGCGAACAGTTTATATAATACTAGTTGTTTTTTGGATTATTATTAATAGAAATTTTATAAATAATATTAAAAAAGAGCATAGACTGGTAGTGACAGCAACGATAATAGTCATCTCTTTACTGGCGTTAACATTTTTTTTCAAACATATAACTAATAATAGTGAATCATATAGCCATCGTGTGTAGGGTGTTGTGA

>CQ1841-Jun2011

AATTTGGGCATCAGAGAGACCGTTGAGTCATAGGTTTTTTTATGCAATTACAATTTTGGTTTCCTATATTCTACTATATGTCAGCAGAAAATATGACGCTAAACATCAGATTGATAGTTTTGTATTATGGTTAGATCTTTTTTTACTTTTAATATCTAATACGCGAACAGTTTATATAATACTAGTTGTTTTTTGGATTATTATTAATAGAAATTTTATAAATAATATTAAAAAAGAGCATAGACTGGTAGTGACAGCAACGACAATAGTCATCTCTTTACTGGCGTTAACATTTTTTTTCAAACATATAATTAATAATAGTGAATCATATAGCCATCGTGTGTAGGTGTTGTGAC

>CQ2896-Jan2012

ATGTCGCGTACCTGAGACCTTTGAGTCATAAAGATTTTTATGCAATTACAATTTTAGTTTCCTATATTCTACTATATGTCAGCAGAAAATATGACGCTAAACATCAGATTGATAGTTTTGTATTATGGTTAGATCTTTTTTTACTTTTAATATCTAATACGCGAACAGTTTATATAATACTAGTTGTTTTTTGGATTATTATTAATAGAAATTTTATAAATAATATTAAAAAAGAGCATAGACTGGTAGTGACAGCAACGATAATAGTCATCTCTTTACTGGCGTTAACATTTTTTTTCAAACATATAACTAATAATAGTGAATCATATAGCCATCGTGTGTAGGGTGTTGTGA

>CQ3599-Jun2012

CAGTAAGCAATTTCCCTCTACAGCACCTGTTCAGCGCAACTATTACAAAGCTATTGTCGTTGCTGTCACTACCAGACTATGCTCTTTTTTAATATTATTTATAAAATTTCTATTAATAATAATCCAAAAAACAACTAGTATTATATAAACTGTTCGCGTATTAGATATTAAAAGTAAAAAAAGATCTAACCATAATACAAAACTATCAATCTGATGTTTAGCGTCATATTTTCTGCTGACATATAGTAGAATATAGGAAACTAAAATTGTAATTGCATAAAAATTTTTATGAGTCAAACCTCCATTAAAACCATATGAAAAGCCAATTCCCTCTACAGAAACTGTTAC

>CQ3930-Sep2012

ACTTGGCTATGGAGGTTTGACTCATAAAAATTTTTATGCAATTACAATTTTAGTTTCCTATATTCTACTATATGTCAGCAGAAAATATGACGCTAAACATCAGATTGATAGTTTTGTATTATGGTTGGATCTTTTTTTACTTTTAATATCTAATACGCGAACAGTTTATATAATACTAGTTGTTTTTTGGATTATTATTAATAGAAATTTTATAAATAATATTAAAAAAGAGCATAGACTGGTAGTGACAGCAACGACAATAGTCATCTCTTTACTGGCGTTAACATTTTTTTTCAAACATATAATTAATAATAGTGAATCATATAGCCATCGTGTGTAGGTGTTGTGA

>CQ7160-Sep2014

AATTGGCTTCATGGAGGTTTGACTCATAAAAATTTTTATGCAATTACAATTTTAGTTTCCTATATTCTACTATATGTCAGCAGAAAATATGACGCTAAACATCAGATTGATAGTTTTGTATTATGGTTGGATCTTTTTTTACTTTTAATATCTAATACGCGAACAGTTTATATAATACTAGTTGTTTTTTGGATTATTATTAATAGAAATTTTATAAATAATATTAAAAAAGAGCATAGACTGGTAGTGACAGCAACGACAATAGTCATCTCTTTACTGGCGTTAACATTTTTTTTCAAACATATAATTAATAATAGTGAATCATATAGCCATCGTGTGTAGGGTGTTGTGA

>CQ7447-Nov2014

AATTGGCTTTATGGAGGTTTGACTCATAAAAATTTTTATGCAATTACAATTTTAGTTTCCTATATTCTACTATATGTCAGCAGAAAATATGACGCTAAACATCAGATTGATAGTTTTGTATTATGGTTGGATCTTTTTTTACTTTTAATATCTAATACGCGAACAGTTTATATAATACTAGTTGTTTTTTGGATTATTATTAATAGAAATTTTATAAATAATATTAAAAAAGAGCATAGACTGGTAGTGACAGCAACGACAATAGTCATCTCTTTACTGGCGTTAACATTTTTTTTCAAACATATAATTAATAATAGTGAATCATATAGCCATCGTGTGT

>CQ8231-May2015

AATTGGTTTATGGAGGTTTGACTCATAAAAATTTTTATGCAATTACAATTTTAGTTTCCTATATTCTACTATATGTCAGCAGAAAATATGACGCTAAACATCAGATTGATAGTTTTGTATTATGGTTGGATCTTTTTTTACTTTTAATATCTAATACGCGAACAGTTTATATAATACTAGTTGTTTTTTGGATTATTATTAATAGAAATTTTATAAATAATATTAAAAAAGAGCATAGACTGGTAGTGACAGCAACGACAATAGTCATCTCTTTACTGGCGTTAACATTTTTTTTCAAACATATAATTAATAATAGTGAATCATATAGCCATCGTGTGTGGGGTGTTGGGA

>CQ8547-Aug2015

AATTGGTTTATGGAGGTTTGACTCATAAAAATTTTTATGCAATTACAATTTTAGTTTCCTATATTCTACTATATGTCAGCAGAAAATATGACGCTAAACATCAGATTGATAGTTTTGTATTATGGTTGGATCTTTTTTTACTTTTAATATCTAATACGCGAACAGTTTATATAATACTAGTTGTTTTTTGGATTATTATTAATAGAAATTTTATAAATAATATTAAAAAAGAGCATAGACTGGTAGTGACAGCAACGACAATAGTCATCTCTTTACTGGCGTTAACATTTTTTTTCAAACATATAATTAATAATAGTGAATCATATAGCCATCGTGTGTGGGGTGTTGGGA

>CQ9318-Apr2016

ATGCGCTTTATGGAGGTTTGACTCATAAAAATTTTTATGCAATTACAATTTTAGTTTCCTATATTCTACTATATGTCAGCAGAAAATATGACGCTAAACATCAGATTGATAGTTTTGTATTATGGTTGGATCTTTTTTTACTTTTAATATCTAATACGCGAACAGTTTATATAATACTAGTTGTTTTTTGGATTATTATTAATAGAAATTTTATAAATAATATTAAAAAAGAGCATAGACTGGTAGTGACAGCAACGACAATAGTCATCTCTTTACTGGCGTTAACATTTTTTTTCAAACATATAATTAATAATAGTGAATCATATAGCCATCGTGTGTGGGGTGTTGTGATG

>CQ8860-Dec2015

ACGTTGGGCTTCATGGAGGTTTGAGTCATAGGATTTTTATGCAATTACAATTTTAGTTTCCTATATTCTACTATATGTCAGCAGAAAATATGACGCTAAACATCAGATTGATAGTTTTGTATTATGGTTGGATCTTTTTTTACTTTTAATATCTAATACGCGAACAGTTTATATAATACTAGTTGTTTTTTGGATTATTATTAATAGAAATTTTATAAATAATATTAAAAAAGAGCATAGACTGGTAGTGACAGCAACGACAATAGTCATCTCTTTACTGGCGTTAACATTTTTTTTCAAACATATAATTAATAATAGTGAATCATATAGCCATCGTGTGTTAGGTGTTGTGA

>CQ8892-Dec2015

CCTATTATTATTATATGTTTGAAAAAAATGTTAACGCCAGTAAAGAGATGACTATTGTCGTTGCTGTCACTACCAGTCTATGCTCTTTTTTAATATTATTTATAAAATTTCTATTAATAATAATCCAAAAAACAACTAGTATTATATAAACTGTTCGCGTATTAGATATTAAAAGTAAAAAAAGATCCAACCATAATACAAAACTATCAATCTGATGTTTAGCGTCATATTTTCTGCTGACATATAGTAGAATATAGGAAACTAAAATTGTAATTGCATAAAAATTTTTATGAGTCAAACCTCCATTAAAACCATATGAAAAGCCAATTCCCTCTACAGAACTGTTACA

>CQ8614-Sep2015

AAATGGGTTATGGAGGTTTGACTCATAAAAATTTTTATGCAATTACAATTTTAGTTTCCTATATTCTACTATATGTCAGCAGAAAATATGACGCTAAACATCAGATTGATAGTTTTGTATTATGGTTAGATCTTTTTTTACTTTTAATATCTAATACGCGAACAGTTTATATAATACTAGTTGTTTTTTGGATTATTATTAATAGAAATTTTATAAATAATATTAAAAAAGAGCATAGACTGGTAGTGACAGCAACGATAATAGTCATCTCTTTACTGGCGTTAACATTTTTTTTCAAACATATAACTAATAATAGTGAATCATATAGCCATCGTGTGTAGGTGTTGTG

>CQ6558-Apr2014

AATTGGCTTCATGGAGGTTTGACTCATAAAAATTTTTATGCAATTACAATTTTAGTTTCCTATATTCTACTATATGTCAGCAGAAAATATGACGCTAAACATCAGATTGATAGTTTTGTATTATGGTTGGATCTTTTTTTACTTTTAATATCTAATACGCGAACAGTTTATATAATACTAGTTGTTTTTTGGATTATTATTAATAGAAATTTTATAAATAATATTAAAAAAGAGCATAGACTGGTAGTGACAGCAACGACAATAGTCATCTCTTTACTGGCGTTAACATTTTTTTTCAAACATATAATTAATAATAGTGAATCATATAGCCATCGTGTGTAGGGTGTTGTGA

**Other serotypes**

**3**

>CQ7231-2014Sep

ATGGGCCTCTATAGTGTTGATGCTTTTGTAGAGAAACCAAAACCAGAAGAAGCGCCTAGCAATTTAGCTATTATTGGACGTTATCTACTTACTCCTGAGATTTTTTCTATATTAGAAACCCAAAAGCCAGGAGCAGGTAATGAAATTCAATTGACAGATGCTATTGATACATTGAATAAGACACAGAGTGTTTTTGCGCGTGAATTTGTGGGCAAACGTTATGATGTTGGTGATAAGTTTAATTTTATGAAAACATCAATTGATTATGCTCTTCAACATCCTCAGATTAAAGAGAGTTTAAAAAATTACGTTATTGCACTTGGTAAGATTTGGAGAAGA

**4**

>CQ4955-2015Jun

TTCTGGTTTTCTTATATCTAATTATGTTCCGTCTATATTTATATGGGTTTGCTTTCTAATTATTTTTCAAATTACTGGTTTTATTTTACAAAAAGTTAGTATATATGATTTTTCTGTATGGTATCTGATTTTATCTTATTTTTTTATGTTTGGATTAATTTTCAATGAGTATATGGGGTTTCAAACAACTCTGCTGTGGAGCCCTAGTAACTTCTATAATAATGAAGAATTATTTCATTCATATATTTTTATAATTTGGATTTTGTTTTGTTATTCTGTAGGCTATTTATTTTTTTATAGTGATGGAAAGGTACATTATCATTCAGAAGTACAAAATTATCAGGAAAATGAAGAGAAAATTTTGTACAATGCGGGTAGGATTTTAACAGGAGTGGGCA

**5**

>CQ3168-2013Mar

TAGGGCTATGATACTTTTTCAATATTGATTATGTTTATCGAGCAGAACGATCTTTCCTGTTTTTTTTATTAATAATAGGTTATGTAATTAGCACTATTGCTTTGTTAGATATAGTTACAGGAGTATCTTATGTCTTTAATGGTTTGTCTCAGCAACTTTATTTGGCTGTGGGAATTCTAGTTTTAGGCTACTGGAATGCGGATGTGATTGTTCATTATTGGAAAATCATTACCGTGACTTTTTTAGGAGCATCTTTGTTGATTTCAGCGGATATTTATTTTCACTATTTTCGAGGACATACTTTTTCAAATATTGATTATTTTATCGAGCA

>CQ7441-2014Nov

TAGGGCTATGATACTTTTTCAATATTGATTATGTTTATCGAGCAGAACGATCATTCCTATTTTTTTTATTAACAATAGGTTGTTTAATTAGCACTATTGCTTTGTTAGATATAGTTACGGGAGTATCTTATGTCTTTAATGGTTTGTCTCAGCAACTCTATTTGGCTGTGGGAATTCTAGTTTTAGGCTACTGGAATGCTGATGTGATTGTTCATTATTGGAAAATCATCACCATGACTTTTTTGGGAGCATGTTTGCTGATTTCAGTGGATATTTATTTTCACTACTTTCAAGGACATACTTTTTCAAATATTGATTATGTTTATCGAGC

**8**

>CQ4748-2013Apr

TTGAGCCTGGAGATGGAGAACTTGAAGACATACGTCAAAAGATATCAAATCTGAATTTGACTGATCATATTACAATATATGATTGGGTTAATCAGAGAGATAAAAAAATACTATTTCAAGCTAATCAGACTTTGATATTGCCTTCTTATAATGAGGGGCTCCCGATGGCAATATTGGAAGCTATGGCTTCTGGATTAGCAATCATATCAACGCCTGTAGGTGGCATACCTGAAATTTACACGAGGATA

**15A**

>CQ5605-2013

AATTGCTATCATCACCACAAAGCTATGATGGATACAATTAGTCTTTCACTTTGCTTATTTTTTCCTCTAGTAATAGTTCCCCGTATGAATAATGACAACAAAAATGAATAAAGCAAAATCACTGAAATAAAACCACCATCTGCAAGCATTTTACCAAGTTCATAAATATAATTAACAGAGTATGAACCTAGTCCTTTCCCAAATATAGCACTAATATAATCATGATAAAATAAGTTGTCCCATAGGAAGGAAATAGTATTTGTTCGTCCCGCAAACTCTGTCCTATTCTCATATGATAATGCCTTTGTAACATTAAAAAAAGTATACAAATTATCAGGTAAAACTATCTCAATGAGTTTATAGGCAATTAAAAAAAGAAGAGATATTCCAGCACTGGTTACTAATAATC

>CQ10492-2016

TTTAACTGCCTATAACTCATTGAGATAGTTTTACCTGATAATTTGTATACTTTTTTTAATGTTACAAAGGCATTATCATATGAGAATAGGACAGAGTTTGCGGGACGAACAAATACTATTTCCTTCCTATGGGACAACTTATTTTATCATGATTATATTAGTGCTATATTTGGGAAAGGACTAGGTTCATACTCTGTTAATTATATTTATGAACTTGGTAAAATGCTTGCAGATGGTGGTTTTATTTCAGTGATTTTGCTTTATTCATTTTTGTTGTCATTATTCATACGGGGAACTATTACTAGAGGAAAAAATAAGCAAAGTGAAAGACTAATTGTATCCATCATAGCTTTTGTGGTGATGATTAGCATTATTGTTTGGAATAGTACGC

**34**

>CQ3916-2012Sep

AAGTATCTTTACTTTACTTTTTTGATAGCAACACTGTTTTGCATTTTAAATGCTGAAGAGTATGCTATTGAGTTCAATGGAGGTACAGTTTTTATTGTGACGATTTCAATGCTCGTTTTTACAATAATAACAGTATTATCAAAGAAAATATTTAAGACTAATACTTTCACCTTTAAGAATAAATTAAAATATCTATATGTCAGTAAAAGTCTAATTTTTACAATAATTATTATTCAGATTTTAAATCTTATTTTCTTTTATCGATACGAACAAGCTTTATTCAGCGCTTATGTTGGCGGTCGTGGAAGTTTCTCGCAAATAATTAATAACTATGACCAATTAGTAAAGTTTTTTACTGAAGATA

>CQ4780-2013Apr

ATTATCTTTACTTTACTTTTTTGATAGCAACACTGTTTTGCATTTTAAATGCTCAAGAGTATGCTATTGAGTTCAATGGAGGTACAGTTTTTATTGTGACGATTTCAATGCTCGTTTTTACAATAATAACAGTATTATCAAAGAAAATATTTAAGACTAATACTTTCACCTTTAAGAATAAATTAAAATATCTATATGTCAGTAAAAGTCTAATTTTTACAATAATTATTATTCAGATTTTAAATCTTATTTTCTTTTATCGATACGAACAAGCTTTATTCAGCGCTTATGTTGGCGGTCGTGGAAGTTTCTCGCAAATAATTAATAACTATGACCAATTAGTAAAGTTTTTTACTGAAGACTTAGTCGGATTGA

**14**

>CQ232-2009Nov

CCGCGATTATTATTGGGGTCCATTCTATGTATATAAGTTTTATTTATAGGACAGGAGTTTTAGGAAGTATAATAATAACAGTAATGTTTTATTATCTGTTTTCTAAATTTTTAAAATGTGATTCATCTGAGAGACTAAGAAGTATTGGCTATATTCTAGCTTTGTCAGTATTTTGGCA

>CQ1025-2010Nov

TGCCGATTATTATTGGGGTCCATTCTATGTATATAAGTTTTATTTATAGGACAGGAGTTTTAGGAAGTATAATAATAACAGTAATGTTTTATTATCTGTTTTCTAAATTTTTAAAATGTGATTCATCTGAGAGACTAAGAAGTATTGGCTATATTCTAGCTTTGTCAGTATTTTGGCA

>CQ1337-2011Feb

GTAGACAGATTATTATTGGGGTCACATTCTATGTATATAAGTTTTATTTATAGGACAGGAGTTTTAGGAAGTATAATAATAACAGTAATGTTTTATTATCTGTTTTCTAAATTTTTAAAATGTGATTCATCTGAGAGACTAAGAAGTATTGGCTATATTCTAGCTTTGTAGTATTTTGGCA

>CQ3518-2012Jun

TGCTGATTATTATTGGGGTCCATTCTATGTATATAAGTTTTATTTATAGGACAGGAGTTTTAGGAAGTATAATAATAACAGTAATGTTTTATTATCTGTTTTCTAAATTTTTAAAATGTGATTCATCTGAGAGACTAAGAAGTATTGGCTATATTCTAGCTTTGTAGTATTTTGGCA

>CQ3590-2012Jun

TGCTGATATTATTGGGGTCCATTCTATGTATATAAGTTTTATTTATAGGACAGGAGTTTTAGGAAGTATAATAATAACAGTAATGTTTTATTATCTGTTTTCTAAATTTTTAAAATGTGATTCATCTGAGAGACTAAGAAGTATTGGCTATATTCTAGCTTTGTCAGTATTTTGGCA

>CQ6633-2014May

AAGGGGATTATTATTGGGGTCCATTCTATGTATATAAGTTTTATTTATAGGACAGGAGTTTTAGGAAGTATAATAATAACAGTAATGTTTTATTATCTGTTTTCTAAATTTTTAAAATGTGATTCATCTGAGAGACTAAGAAGTATTGGCTATATTCTAGCTTTGTCAGTATTTTGGCC

>CQ6788-2014Jun

AGCCTGATATTATTGGGGTCCATTCTATGTATATAAGTTTTATTTATAGGACAGGAGTTTTAGGAAGTATAATAATAACAGTAATGTTTTATTATCTGTTTTCTAAATTTTTAAAATGTGATTCATCTGAGAGACTAAGAAGTATTGGCTATATTCTAGCTTTGTAGTATTTTGGCC

>CQ6810-2014Jun

GTGCTGATTATTATTGGGGTCCATTCTATGTATATAAGTTTTATTTATAGGACAGGAGTTTTAGGAAGTATAATAATAACAGTAATGTTTTATTATCTGTTTTCTAAATTTTTAAAATGTGATTCATCTGAGAGACTAAGAAGTATTGGCTATATTCTAGCTTTGTAGTATTTTGGC

>CQ9281-2016Apr

TAGGGTGATTTTATTGGGGTCCATTCTATGTATATAAGTTTTATTTATAGGACAGGAGTTTTAGGAAGTATAATAATAACAGTAATGTTTTATTATCTGTTTTCTAAATTTTTAAAATGTGATTCATCTGAGAGACTAAGAAGTATTGGCTATATTCTAGCTTTGTCAGTATTTTGGCA

>CQ1622-2011Apr

TAACGTGATTATTATTGGGGTCACATTCTATGTATATAAGTTTTATTTATAGGACAGGAGTTTTAGGAAGTATAATAATAACAGTAATGTTTTATTATCTGTTTTCTAAATTTTTAAAATGTGATTCATCTGAGAGACTAAGAAGTATTGGCTATATTCTAGCTTTGTAGTATTTTGGCA

**22F**

>CQ854-2010Sep

CCAGTAGTCTTCCAGCGTATGCTGGAGAATGGCCCATACTATAATCTATTTAAGATTCCCATTATTGAACCTATATACGATTTTCTTTTTGTAGAAATGCTCAATAAGAGAACCAAAACAGTTTTCTGCAAGACAGAAAAAGCAGAGTGTTATCTTAAAAACAAAGGATTCAATGATTGTAAAGTAGTGGGCGTAGGTCTAGATATTGAGAAGTTTGAACAAGAAGAGGAACCTACAGAGGATACTATAGAGCTTTTAAGAAGAATGGAGAATAAACAAAATATTCTTTATGTAGGTTCACTATCAAAAAGAAAAAACACAGCTCAGTTAATTAGAATTTTTAATATTTTAAAATCTAAAAGTGGTAAGAAAAACGAACTTCAATTGGTATTGATTGGTAAGGATGAGGATAATATTGTTGAAAAAATTAACTATTCACGGTTTAAAGATGATATTATTTATCAACCTTATTTAAAGAATTCTCAGCTTCAATTTATTTACCCATCATCACAACTACTTGTGCTCCCGTCAGTTCAAGAGATTTTTGGTATGGTATTACTTGAGGCAATGTATTTTAAGTTGTCTGTTGTTTCCAGCGCAGGTGGCTGGAGA

>CQ8840-2015Dec

TTGTAGTCTCAGCGCGTGTGCTGGAGGTTGTCTGTTGTTTCAGCGCAAGTGCTGGAGAATTATTGAACCTATATACGATTTTCTTTTTGTAGAAATGCTCAATAAGAGAACCAAAACAGTTTTCTGCAAGACAGAAAAAGCAGAGTGTTATCTTAAAAACAAAGGATTCAATGATTGTAAAGTAGTGGGCGTAGGTCTAGATATTGAGAAGTTTGAACAAGAAGAGGAACCTACAGAGGATACTATAGAGCTTTTAAGAAGAATGGAGAATAAACAAAATATTCTTTATGTAGGTTCACTATCAAAAAGAAAAAACACAGCTCAGTTAATTAGAATTTTTAATATTTTAAAATCTAAAAGTGGTAAGAAAAACGAACTTCAATTGGTATTGATTGGTAAGGATGAGGATAATATTGTTGAAAAAATTAACTATTCACGGTTTAAAGATGATATTATTTATCAACCTTATTTAAAGAATTCTCAGCTTCAATTTATTTACCCATCATCACAACTACTTGTGCTCCCGTCAGTTCAAGAGATTTTTGGTATGGTATTACTTGAGGCAATGTATTTTAAGTTGTCTGTTGTTTCCAGCGAG

>CQ1007-2010Oct

GAGGCAATCATTCCATGTATATATTTATAATGGTCCATACTATAATCTATTTAAGATCCCCATTATTGAATCTATGTACGATTTTCTTTTTGTAAGAATGCTCAATAAGAGAACCAAGACAGTTTTCTGCAAGACAGAAAAGGCAAAGCATTATCTTAAAAATAAGGGATTCAATGATTGTAAAGTAGTGGGCGTAGGTCTAGATGTTGAAAAGTTTGAACAAGAAGAGGAACCTACGGAGAATACTATAGAACTTTTAAAAAGAATGGAGAATAAGCAAAATATTCTTTATGTAGGCTCACTATCAAAAAGAAAAAACACAGCTCATTTAATTAGAATTTTTAATATTTTAAAATCTAAAAGTGGTAAGAAAAACGAACTTCAATTGGTATTGATTGGTAAGGATGAGGGTAATATTGTTGAAAAAATTAACTATTCACGGTTTAAAGATGATATTATTTATCAACCTTATTTAAAGAACTCTCAGCTTCAATTTATTTACCCATCATCACAACTATTTGTGCTCCCGTCGATTCAAGAGATTTTTGGTATGGTATTACTTGAGGCAATGTATTTTAAGTTGTCTGTTGTTTCCAGCGCAGTTGCTGGAGA

**35B**

>CQ7559-2014Nov

AAGGTAGGTATTTACCACATCCAATGTACGGAGTGCGTGTTGCAAATTTTGCAATTCCTTTATTGAAAAAGTATAAAAATATAAAATTTATTTCAGTAATACATGATTTAGAATCGTTAAGAAAAGGAATCCAAGGAGTCATTGAAGATAATGAAACAACAAATGCTATTGCAGACAAAGAGTTGTTGTCAAAATTTGATAAAGTGATTTCCCATAATCCGAAAATGACAGAATATTTAGAAGGGATAGGGATAAAAAAAGAAAATTTAGTTGAATTGCAAATATTCGACTACCTAGATCCATCAGAAATAGAAGAAAAGATTGAAGATGGAGTGGTTATAGCGGGGAATCTAGCAAAAGGAAAAAGTTCATATATATATAAGTTGTTAGAAAATGAACTGAACTTCAAATTAAATCTTTTTGGGCCAAATTTTATTAACGAAGAGCTACCAGAAAACGTTGAGTATTTTGGTAGTTTACCTCCAAATAAATTGCCTCAAAAGCTAGTAGGTAAGTTTGGTTTGGTTTGGGACGGTGATAGTCTAGAAACTTGTAGTGGAAATACTGGTAACTATTTGAAGTATAATAATCCACATAAAACCTCATTATATCTTGCTTCAGGAATACCTGTAAATT

**35F**

>CQ8466-2015Jul

ATGAGAGACTTTTACTCGTTCTAGGAAAAGGCTCCTAGTAATTATTGGCTGTATGATGAAACGGATTCTCGATTAACATTTTATAGTTCCTGTATATTGTTGATATGCAGTTTATTAATAAGATGGATCCCAGAACTATTCTCTAAGTTCGGATATATATTTAAAGCTTTTAAACTCACCTTTATTATAAATGCAGTTGTTAGTTTTTGGGTCTCCTTTACCTATCTCAATTTGAGTCATTCTTATATCAATAATCTTTTTTTAAAATTAGACTATATATTAGGTGGTCGTATATACTTGATGAATAAATCGTTGAATTTGTATGGTTTTGGATTATTCGGACGACCAGTTGAATGGAATGGAAATGGTCTGACCATTGAAGGAGTTAGAAACTATCAGACCTATCTGTATGTTGATAATTTGTATGTTCAAATTTTACAAAAATTTGGTTTACTCGCTCTAGGAATAATGCTCCTAGTCAAC
